# Fragment Length of Circulating Tumor DNA

**DOI:** 10.1371/journal.pgen.1006162

**Published:** 2016-07-18

**Authors:** Hunter R. Underhill, Jacob O. Kitzman, Sabine Hellwig, Noah C. Welker, Riza Daza, Daniel N. Baker, Keith M. Gligorich, Robert C. Rostomily, Mary P. Bronner, Jay Shendure

**Affiliations:** 1 Department of Pediatrics, Division of Medical Genetics, University of Utah, Salt Lake City, Utah, United States of America; 2 Department of Radiology, University of Utah, Salt Lake City, Utah, United States of America; 3 Department of Neurological Surgery, University of Washington, Seattle, Washington, United States of America; 4 Department of Genome Sciences, University of Washington, Seattle, Washington, United States of America; 5 Department of Human Genetics, University of Michigan, Ann Arbor, Michigan, United States of America; 6 ARUP Laboratories, Salt Lake City, Utah, United States of America; 7 Department of Pathology, University of Utah, Salt Lake City, Utah, United States of America; Brigham and Women's Hospital, UNITED STATES

## Abstract

Malignant tumors shed DNA into the circulation. The transient half-life of circulating tumor DNA (ctDNA) may afford the opportunity to diagnose, monitor recurrence, and evaluate response to therapy solely through a non-invasive blood draw. However, detecting ctDNA against the normally occurring background of cell-free DNA derived from healthy cells has proven challenging, particularly in non-metastatic solid tumors. In this study, distinct differences in fragment length size between ctDNAs and normal cell-free DNA are defined. Human ctDNA in rat plasma derived from human glioblastoma multiforme stem-like cells in the rat brain and human hepatocellular carcinoma in the rat flank were found to have a shorter principal fragment length than the background rat cell-free DNA (134–144 bp vs. 167 bp, respectively). Subsequently, a similar shift in the fragment length of ctDNA in humans with melanoma and lung cancer was identified compared to healthy controls. Comparison of fragment lengths from cell-free DNA between a melanoma patient and healthy controls found that the *BRAF* V600E mutant allele occurred more commonly at a shorter fragment length than the fragment length of the wild-type allele (132–145 bp vs. 165 bp, respectively). Moreover, size-selecting for shorter cell-free DNA fragment lengths substantially increased the *EGFR* T790M mutant allele frequency in human lung cancer. These findings provide compelling evidence that experimental or bioinformatic isolation of a specific subset of fragment lengths from cell-free DNA may improve detection of ctDNA.

## Author Summary

During cell death, DNA that is not contained within a membrane (i.e., cell-free DNA) enters the circulation. Detecting cell-free DNA originating from solid tumors (i.e., circulating tumor DNA, ctDNA), particularly solid tumors that have not metastasized, has proven challenging due to the relatively abundant background of normally occurring cell-free DNA derived from healthy cells. Our study defines the subtle but distinct differences in fragment length between normal cell-free DNA and ctDNA from a variety of solid tumors. Specifically, ctDNA was overall consistently shorter than the fragment length of normal cell-free DNA. Subsequently, we showed that a size-selection for shorter cell-free DNA fragments increased the proportion of ctDNA within a sample. These results provide compelling evidence that development of techniques to isolate a subset of cell-free DNA consistent with the ctDNA fragment lengths described in our study may substantially improve detection of non-metastatic solid tumors. As such, our findings may have a direct impact on the clinical utility of ctDNA for the non-invasive detection and diagnosis of solid tumors (i.e., the “liquid biopsy”), monitoring tumor recurrence, and evaluating tumor response to therapy.

## Introduction

Increased quantity of cell-free DNA in the circulation has been associated with malignant solid tumors [1]. Longitudinal studies have reported reductions in cell-free DNA quantity in response to therapy and elevations associated with recurrence suggesting quantification of cell-free DNA may be useful for monitoring disease status [2–4]. However, quantifying cell-free DNA as a marker of disease and its extent has been limited. The quantity of cell-free DNA has not correlated well with stage and histological subtype [5, 6]. In addition, large inter-subject variations of cell-free DNA quantification have been described leading to overlap between malignant disease, benign tumors, and healthy controls [7, 8]. Moreover, increased quantity of cell-free DNA is non-specific to cancer and has been associated with other conditions such as autoimmune disease and environmental exposures [9, 10]. Finally, except in patients with advanced metastatic disease, tumor-derived cell-free DNA (i.e., circulating tumor DNA, ctDNA) forms only a small minority of the cell-free DNA in circulation against a background of fragments mostly derived from normal cells. Therefore, the quantification of cell-free DNA alone is of little prognostic value.

As an alternative, detecting specific variants or mutational hotspots in ctDNA may have important clinical implications in the shift towards personalized medicine for diagnosing and/or monitoring malignancies. In lung cancer, *EGFR* mutations in ctDNA have been associated with prognosis and utilized for determining therapy (e.g., activating mutations that confer sensitivity to tyrosine kinase inhibitors) [11]. However, molecular ctDNA studies in a variety tumor types have largely focused on advanced or metastatic disease in which ctDNA is more readily detectable compared to localized disease [12]. Bettegowda et al. reported a substantial reduction in detectability of ctDNA in localized disease compared to metastatic tumors for breast, colon, pancreas, and gastroesophageal cancers [13]. Moreover, ctDNA from glioblastoma multiforme (GBM), a primary brain tumor associated with neovascularization and disruption of the blood-brain barrier, was undetectable [13]. This latter finding supports the general perception that detection of ctDNA from non-metastatic solid tumors is particularly challenging since GBM does not metastasize beyond the central nervous system.

Emerging approaches to improve detection of ctDNA include amplicon-based strategies in colorectal cancer [14] and integrated digital error suppression during deep sequencing in lung cancer [15]. While the latter methods seek to eliminate artifacts during sequencing to improve bioinformatic analytic sensitivity of mutant allele detection, the former techniques exploit apparent size differences between ctDNA and cell-free DNA. Specifically, previous amplicon-based studies have shown that ctDNA is highly fragmented and occurs most commonly at a size <100 bp, while normal cell-free DNA is proportionally more represented at a size >400 bp [16]. In this study, we initially sought to determine the feasibility of detecting ctDNA associated with GBM by utilizing a xenograft tumor model to exploit genomic species differences to separate ctDNA from the background host animal benign cell-free DNA. In so doing, we identified precise differences in fragment lengths between ctDNA and normal cell-free DNA, which were more narrow and more consistent than previously described [16, 17]. In addition, we found strong evidence of a 10 bp periodicity in ctDNA that was less prominent in normal cell-free DNA. These observations led us to explore if similar findings were present in tumors outside the brain and subsequently translated to cell-free DNA samples obtained from cancer patients. Collectively, the results described herein demonstrate that the fractional selection of cell-free DNA with a specific size range that is 20–50 bp shorter than the size of normal healthy cell-free DNA may substantially enrich for ctDNA in human cancer.

## Results

### GBM Xenograft Model

**Fig 1:**
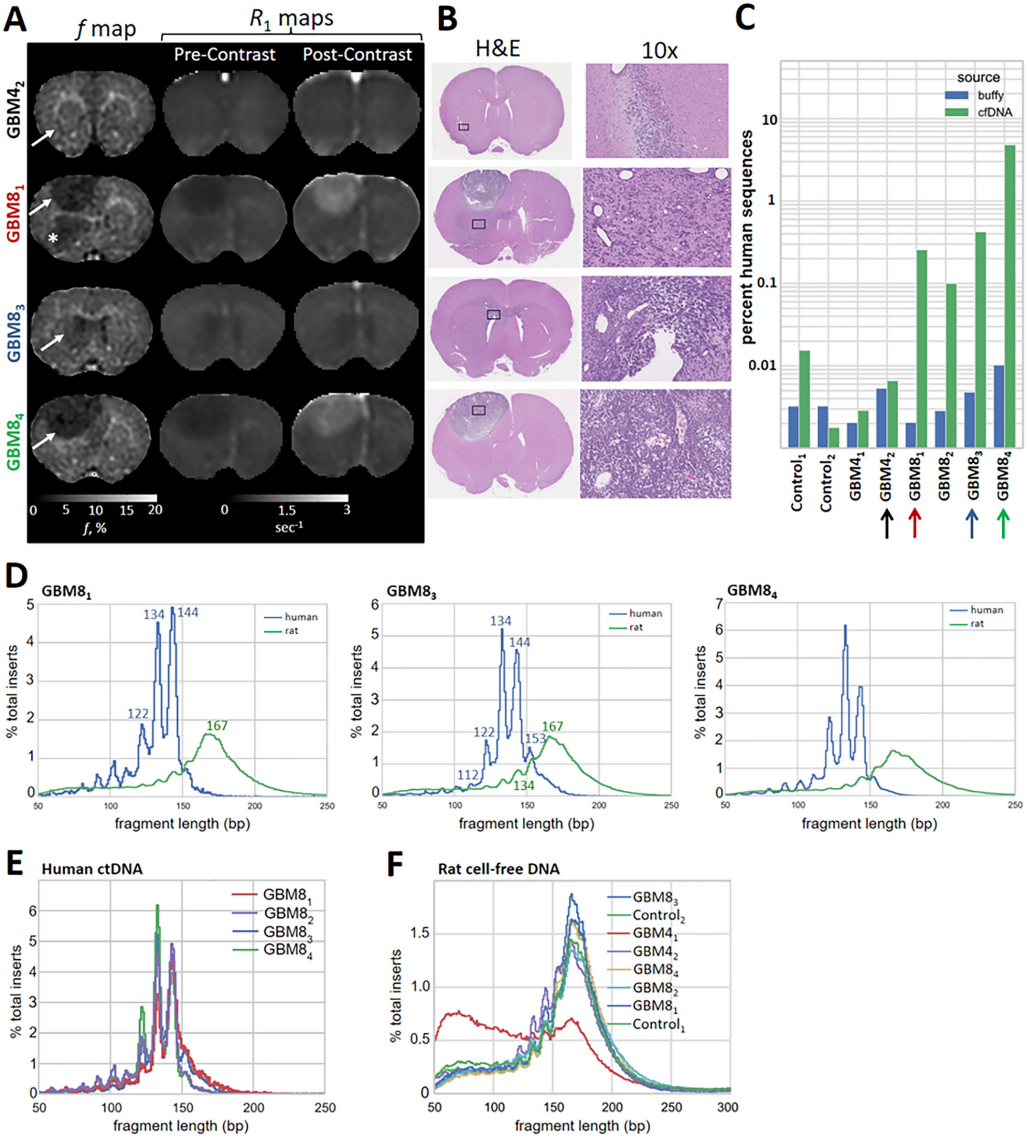
Periodicity and shorter fragment length of ctDNA derived from GBM8. In **A**, coronal *f* maps and pre- and post-contrast *R*_1_ maps with matched histology (**B**) and percent of human ctDNA detected in rat plasma (**C**, colored arrows identify results that correspond to images in **A**). GBM4_2_ is a small tumor (**A**, white arrow) confirmed on histology (**B**, black box) with no evidence of a disrupted blood-brain barrier (i.e., post-contrast enhancement on *R*_1_ maps; **A**). In GBM8_1_, a large tumor (white arrow on *f* map) is associated with disruption of the blood-brain barrier above the corpus callosum, but not below (asterisk on *f* map). GBM8_3_ is an infiltrating tumor (white arrow) with no evidence of blood-brain barrier disruption, but possible invasion into the ventricle as identified on histology (**B**, black box). GBM8_4_ is a large bulky tumor with disruption of the blood-brain barrier (**A**). Human ctDNA was detected at a level above the control animals for all GBM8 tumors (**C**). Fragment length distribution for rat cell-free DNA (green line) and human ctDNA (blue line) inferred from paired-end sequencing are plotted in **D**. All detected ctDNA demonstrated the same strong periodicity and shorter fragment length compared to rat cell-free DNA (**E**). Distribution of normal rat cell-free DNA was largely consistent between animals (**F**).

Established human GBM stem-like cell lines (GBM4 and GBM8) [18, 19] were implanted in the nude rat brain. Control animals underwent an identical surgical procedure and were inoculated with medium only. Quantitative magnetic resonance imaging techniques were implemented on a 3T whole-body clinical scanner (Philips Achieva) to phenotype the tumors. Fast bound-pool fraction imaging (FBFI), a method validated with histology to measure myelin density and identify tumor associated disruption of normal brain tissues [20], was used to produce bound-pool fraction maps (*f* maps) to detect and differentiate between bulky and infiltrative lesions. The variable flip angle method [21] was used to measure *T*_1_ relaxivity (*R*_1_ maps, where *R*_1_ = 1/*T*_1_) before and after administration of gadolinium (gadopentetate dimeglumine, Bayer HealthCare), an intravenous contrast agent that shortens *T*_1_ and identifies disruption of the blood-brain barrier (i.e., hyperintense signal on post-contrast *R*_1_ maps relative to pre-contrast *R*_1_ maps). Our initial experiments found that GBM4 yielded small, focal, non-enhancing lesions (Fig 1A and 1B, S1A and S1B Fig). In contrast, GBM8 produced heterogenous lesions that ranged from large, well-circumscribed tumors with strong contrast enhancement (i.e. disruption of the blood-brain barrier) to infiltrative lesions with absent or minimal contrast enhancement (Fig 1A and 1B, S1A and S1B Fig).

### Characteristics of ctDNA Associated with GBM

Detection of human ctDNA associated with GBM4 was not greater than the control animals (Fig 1C), which was attributable to the small tumor size and absence of blood-brain barrier disruption. Human ctDNA was detected in all animals implanted with GBM8 including infiltrative lesions with absent (e.g., GBM8_3_, Fig 1A and 1B) to minimal (e.g., GBM8_2_, S1A and S1B Fig) disruption of the blood-brain barrier (Fig 1C, S1C Fig). The percent human ctDNA in the buffy coat, where some residual plasma remains present, appeared correlated with the fraction in plasma, but much lower, indicating that neither intact tumor cells nor a high molecular weight fraction of ctDNA were present in the circulation (Fig 1C). Unexpectedly, there was a precise difference in fragment length between human ctDNA and rat cell-free DNA. The most common fragment lengths in human ctDNA were 134 bp and 144 bp (Fig 1D, S1D Fig), which was in contrast to the most common fragment length of 167 bp in rat cell-free DNA (Fig 1F). Human ctDNA fragment lengths also exhibited a strong ~10 bp periodicity that was not as evident in the rat cell-free DNA. This pattern was consistent across all animals where human ctDNA was detected (Fig 1E).

**Fig 2:**
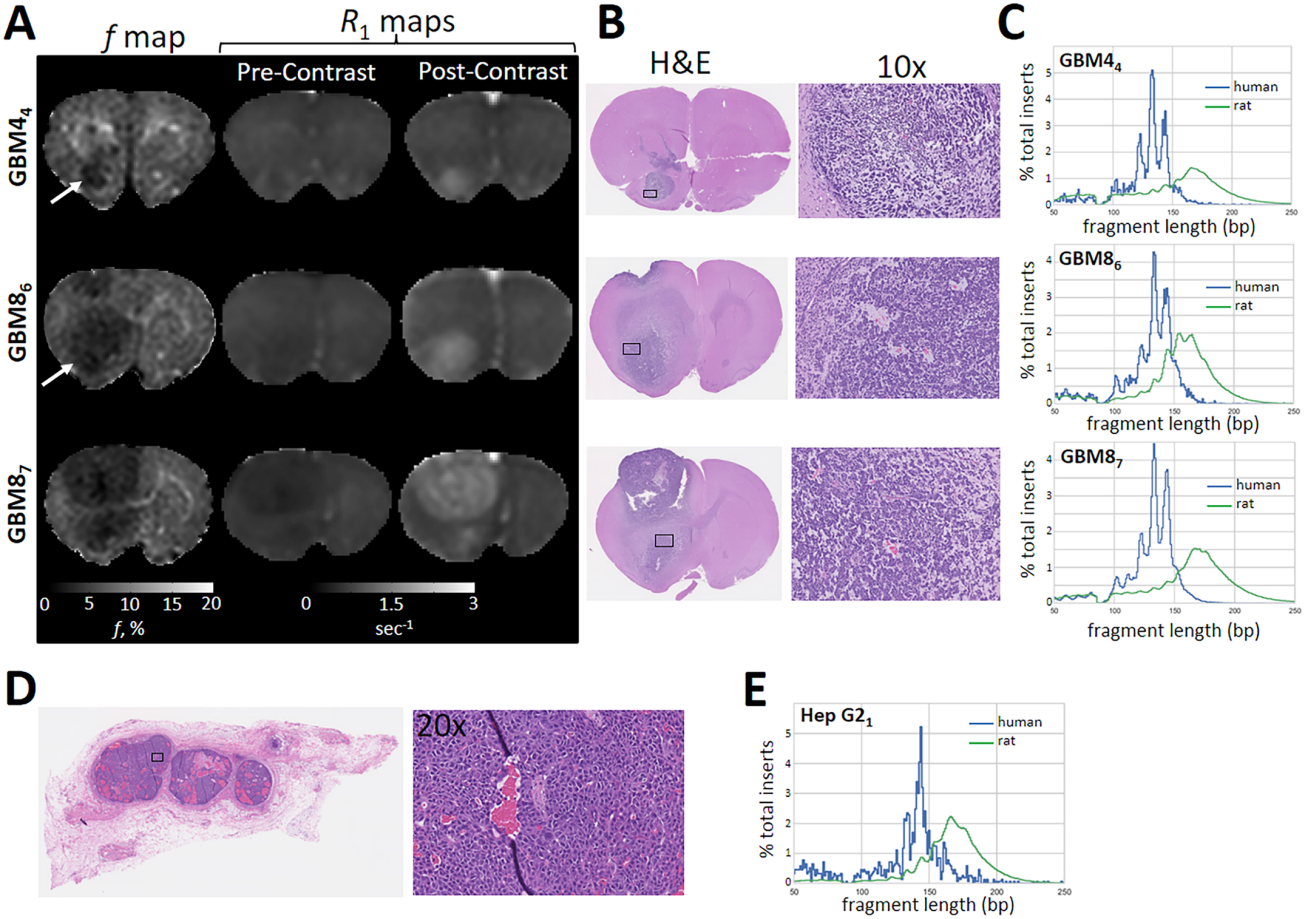
The ctDNA from GBM4 and hepatocellular carcinoma have a similar shortening of fragment length as GBM8. GBM4_4_ is a small tumor (**A**, white arrow) confirmed on histology (**B**, black box) with evidence of a disrupted blood-brain barrier by presence of contrast enhancement on post-contrast *R*_1_ maps. The fragment length and periodicity seen previously in GBM8 (Fig 1) are also present in GBM4 (**C**), which also replicates in new animals with GBM8, as shown in GBM8_6_ and GBM8_7_. Histology from an animal implanted with human hepatocellular carcinoma (Hep G2 cells) in the flank identified a highly vascular tumor (**D**). The ctDNA from human hepatocellular carcinoma had a similar fragment length (**E**) that was seen in the GBM tumors suggesting that the observed differences in fragment length were not secondary to effects of the blood-brain barrier or specific to GBM.

To determine if the fragment length and periodicity extended beyond the GBM8 cell line, nude rats were again implanted with either GBM4 or GBM8. Animals implanted with GBM4 were serially imaged until the presence of blood-brain barrier disruption was evident (i.e., contrast-enhancement on MRI) or animals lost more than 10% bodyweight. As before, GBM8 animals developed tumors that were large and exhibited a range of phenotypes (Fig 2A and S2 Fig). Fragment length and periodicity was consistent with that present in the initial experiment (Fig 2C). After a post-surgical interval nearly twice as long as GBM8, GBM4 tumors developed (Fig 2 and S2 Fig). GBM4 tumors tended to grow more anteriorly towards the olfactory bulbs, which led to weight loss in animals before tumor size was similar to GBM8. In a single animal implanted with GBM4, ctDNA was adequately detected and a similar fragment length and periodicity as seen with GBM8 was identified (Fig 2C).

### Detection of ctDNA in a Xenograft Model of Hepatocellular Carcinoma

To evaluate the role of both GBM and the blood-brain barrier in determining fragment length and periodicity, human hepatocellular carcinoma cells (Hep G2) were implanted subcutaneously in the flank of three nude rats. A palpable tumor (approximately 10 mm at maximal diameter), confirmed with histology, formed in a single animal (Fig 2D). The fragment length of ctDNA was consistent with that described in GBM4 and GBM8 (Fig 2E). There was evidence for a similar periodicity, but the relatively low amount of detected ctDNA may have contributed to a noisier distribution of fragment size. Regardless, the replication of results in a xenograft model of hepatocellular carcinoma suggested that the periodicity and reduced fragment length of ctDNA may be general properties of ctDNAs in cancers beyond GBM.

### Characteristics of Cell-Free DNA and ctDNA in Human Melanoma

**Fig 3:**
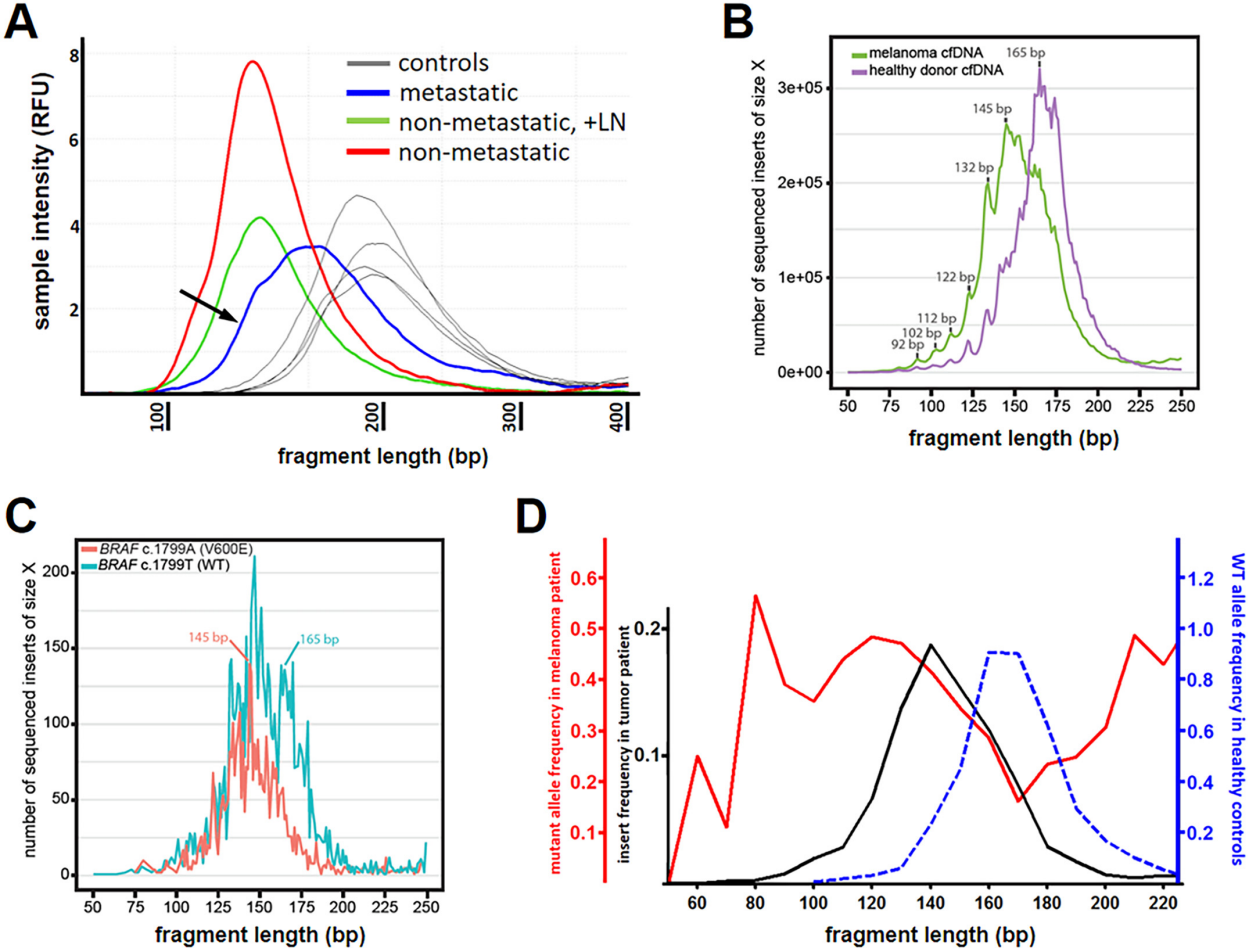
The cell-free DNA and ctDNA from melanoma patients consisted of shorter fragments than the healthy controls and the WT allele fragments. In **A**, the relative fragment length of cell-free DNA obtained from melanoma patients with and without metastatic disease (± lymph node, LN; **A**) tended to be shorter by densitometry compared to cell-free DNA from four healthy controls (**A**, gray lines). In **B**, the fragment lengths derived from cell-free DNA deep-sequencing in a patient with melanoma (**A**, black arrow) were generally shorter than the fragment lengths present in the pool of healthy controls (green and purple lines, respectively). (**C)** In the melanoma patient, cell-free DNA fragment lengths containing the mutant allele (*BRAF* V600E, red line) were shorter compared to the fragment lengths containing the wild-type (WT) allele (blue line). In the shorter fragments there was general overlap between the mutant and WT allele sizes since the *BRAF* V600E mutation is heterozygous. **(D)** Fragment lengths between 110–140 bp had the highest proportion of the mutant allele (**D**, red solid line; the mutant allele frequency <100 bp was erratic due to few observations). In **D**, the solid black line represents the overall frequency for each range of fragment lengths in the melanoma patient and indicates that there may be insufficient amount of DNA for detecting mutant alleles below 100 bp. Of note, the WT allele from the healthy control occurred more commonly between 160–180 bp (**D**, blue dashed line).

We next considered the effects of the xenograft model on ctDNA fragment length and periodicity and sought to determine if evidence of the observed differences in fragment length were present in other types of solid tumors such as melanoma. In contrast to the xenograft models, the cell-free DNA from tumor patients represented an indistinguishable mix of both ctDNA and cell-free DNA derived from normal healthy cells. By densitometry (TapeStation 2200), the cell-free DNA from melanoma patients had globally shorter fragment lengths compared to healthy controls (Fig 3A).

Potential differences in fragment length size between tumor patients and healthy controls were explored by sequencing the cell-free DNA from a melanoma patient with an elevated concentration of cell-free DNA (36.4 ng/mL plasma; Fig 3A, black arrow) to obtain a large sample for comparison to sequencing results from a pooled sample of control cell-free DNA. The most common fragment length in the melanoma patient was shorter than the most common fragment length in the control sample (145 bp vs. 165 bp, respectively; Fig 3B). There was also evidence for more pronounced fragment length periodicity in the cell-free DNA from the melanoma patient (Fig 3B). In the melanoma patient cell-free DNA, the *BRAF* V600E allele frequency was increased at shorter fragment lengths compared to the WT allele (Fig 3C). Of note, the broad distribution of the WT allele (Fig 3C, blue line) included a substantial proportion of overlapping fragment sizes with the mutant allele since the *BRAF* V600E mutation is heterozygous and tumor cells also introduced shorter fragment lengths into the circulation with the WT allele. Subsequently, fragment length for the melanoma patient and the healthy volunteer were binned (e.g. 50 = 50–59 bp; 60 = 60–69 bp, etc.) and the frequency of mutant allele and WT allele was determined, respectively. For a given fragment length, the proportion of the V600E *BRAF* allele to the WT allele was highest in the 110–140 bp fragment length, which was in contrast to the WT allele in the pooled healthy control sample that occurred at the highest frequency between 160–180 bp (Fig 3D). Importantly, there were limited observations of ctDNA fragments <100 bp in the melanoma patient (Fig 3D, black line), which were likely present but not well recovered by current approaches to library preparation [22]. These collective findings indicated an overall shortening of ctDNA fragment size relative to cell-free DNA that was not an effect of the xenograft model, but rather inherent to ctDNAs across different tumor types.

### Characteristics of Cell-Free DNA and ctDNA in Human Lung Cancer

**Fig 4:**
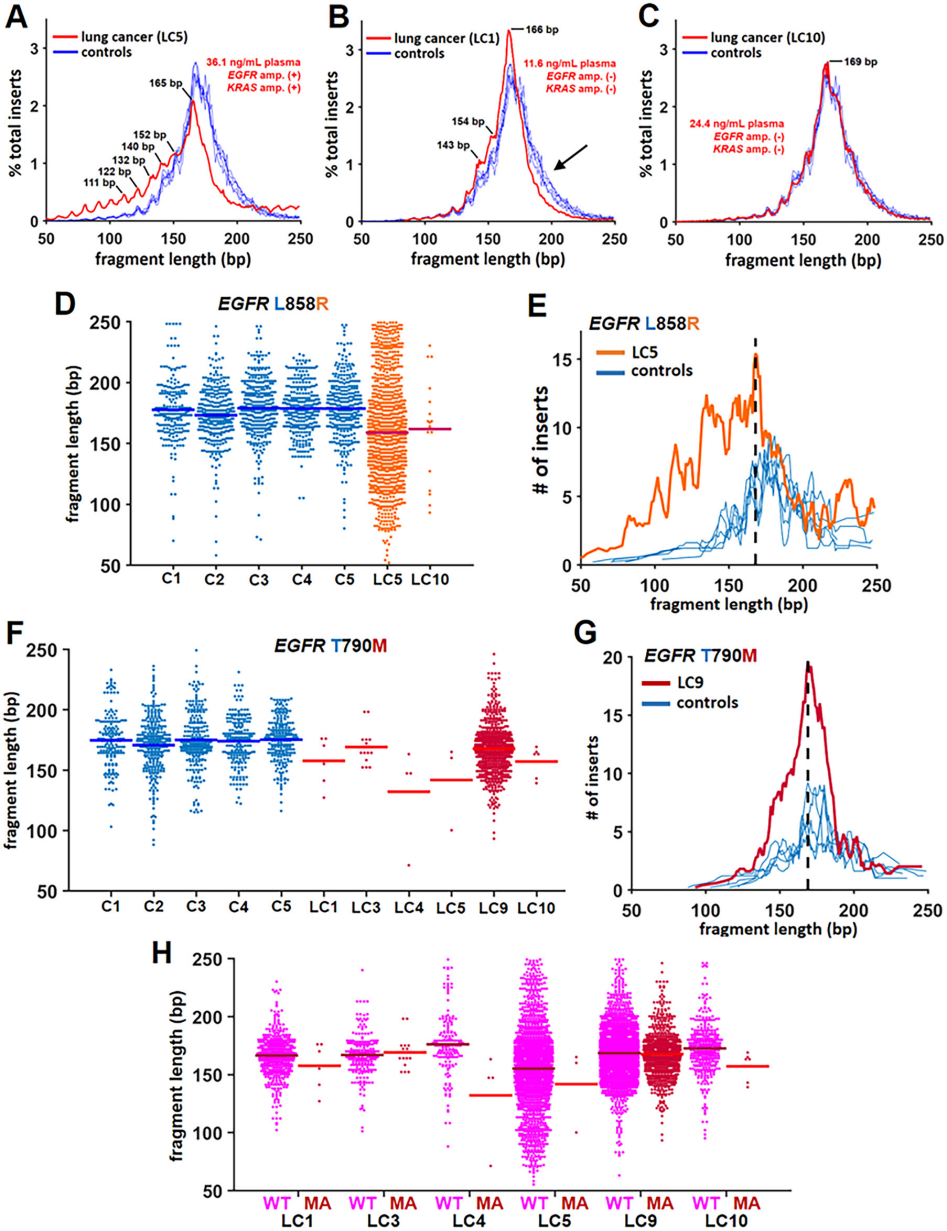
In lung cancer patients, mutant alleles occurred more commonly in shorter fragments of cell-free DNA. In **A-C**, histograms of overall cell-free DNA fragment length from the entire 16-gene capture panel determined by sequencing compared between five healthy controls (blue lines) and individual tumor patients (red line). Plasma concentration of cell-free DNA and presence (+) /absence (-) of *EGFR* and *KRAS* amplifications are also described for each tumor patient. There was a strong left shift (i.e., shorter fragment lengths) and periodicity in LC5 compared to controls (**A**). In **B**, there was a subtle shift towards shorter fragment length that was most apparent at longer lengths (black arrow) where fewer inserts from the tumor patient (LC1) were present compared to the healthy controls. In **C**, no difference between the tumor patient (LC10) and the healthy controls was observed. In **D**, the length of fragments containing the WT or mutant *EGFR* allele is shown for healthy controls (blue dots) and tumor patients with the mutant L858R allele (orange dots). The solid bars indicate the mean fragment length for each sample. In **E**, a histogram of the fragment lengths of the mutant L858R allele from LC5 (orange line) vs. the WT allele in healthy controls (blue lines) demonstrates a higher prevalence of mutant allele at shorter fragment lengths. The black dashed-line identifies the fragment length that corresponds to the most inserts in the tumor patient. Note that the mutant allele more commonly occurs at shorter fragment lengths while the WT allele in healthy controls occurs more commonly at longer fragment lengths. In **F**, the fragment length associated with *EGFR* for the WT allele in the healthy controls (blue dots) and tumor patients with the mutant T790M allele (red dots) is displayed. The solid bars correspond to mean fragment length for each sample. In **G**, a histogram of the fragment length of the mutant allele (L858R) from LC9 (red line) vs. the WT allele in healthy controls (blue lines) is shown. The black dashed-line identifies the fragment length that corresponds to the most inserts in the tumor patient. Note that the WT allele in healthy controls more commonly occurs at longer fragment lengths. In **H**, the *EGFR* fragment length associated with the WT allele (pink dots) and the mutant T790M allele (MA; red dots) in each of the tumor patients are depicted. The mutant allele more commonly occurred at a shorter fragment length compared to the length of the WT allele within the same patient.

We then sought to characterize tumor-related differences in cell-free DNA and ctDNA associated with human lung cancer. A comparison of cell-free DNA from 15 lung cancer patients and 9 healthy controls found a statistically significant difference in plasma concentration of cell-free DNA (31.0±23.3 vs. 11.3±4.6 ng mL/plasma, *p* = 0.006; respectively); however, the range of concentration in tumor patients was broad and overlapped with the concentrations present in healthy controls (S3 Fig). Libraries made with duplex truncated molecular barcoded adapters, which added ~99 bp to each strand of cell-free DNA, were created to enable loading of a consistent concentration (2 ng/μL) of each sample and clear identification of upper/lower markers for direct comparison of densitometry data (TapeStation 2200; S4 Fig). Consistent with our observation in melanoma patients, peak fragment length by densitometry was significantly shorter compared to healthy controls (277.0±4.7 vs. 283.7±4.1 bp, *p* = 0.002; respectively; S3B Fig) indicating a global shift towards smaller fragments in lung cancer patients. However, there was also considerable overlap of peak fragment length between tumor patients and controls (S3B Fig). There was not an association between peak fragment length by densitometry and plasma concentration of cell-free DNA in the lung cancer patients (Pearson’s *r* = –0.20, *p* = 0.47; Fig 4C) or the healthy controls (Pearson’s *r* = 0.19, *p* = 0.63; S3C Fig).

For a subset of samples (tumor, *N* = 7; control *N* = 5), cell-free DNA was converted to Illumina sequencing libraries and enriched for cancer-relevant genes using a 16-gene capture panel. Utilizing fragment lengths from the complete 16 gene capture panel, we observed that the cell-free DNA from tumor patients ranged from a shorter fragment length with (Fig 4A) and without (Fig 4B and S5A–S5D Fig) a strong periodicity to indistinguishable (Fig 4C and S5E–S5G Fig) in fragment length distribution from healthy controls. Cell-free DNA from the tumor patients did not exhibit a fragment length larger than the controls (S5 Fig), which was consistent with densitometry (S3B Fig).

Cell-free DNA from two lung cancer patients (LC5 and LC10) contained the classic *EGFR* L858R mutation [23]. Fragments containing the mutant allele, which originate from the tumor rather than from breakdown of normal cells, were shorter than those bearing the WT allele in healthy controls (Fig 4D). This difference was especially pronounced in one sample (LC5; Fig 4E) with a relatively high mutant allele frequency (74.6%; likely due to *EGFR* amplification, S6 Fig).

Cell-free DNA from six of the lung cancer patients contained the *EGFR* T790M mutation. In 5 out of 6 patients, the mutant allele frequency was relatively low (0.2–6.6%). However, the general trend in these samples was for mutant alleles to occur at shorter fragment lengths (Fig 4F). In one sample (LC9) with a relatively high mutant allele frequency (25.1%; most likely due to an EGFR amplification, S5 Fig) the mutant allele more commonly occurred at shorter fragment lengths compared to the fragment lengths from healthy controls (Fig 4G). The distribution of fragment lengths of the *EGFR* WT allele between tumor patients and healthy controls largely reflected differences seen in S5 Fig, although noisier due to fewer total reads (S7 Fig). Within each lung cancer patient with the mutant T790M allele, comparison of the distribution of the *EGFR* WT allele and the mutant T790M allele fragment lengths identified a general trend for the mutant allele to occur more commonly at shorter fragment lengths (Fig 4H). As with the melanoma patient (Fig 4C), fragment length analysis of the WT allele from tumor patients included an indistinguishable mixture of ctDNA and normal cell-free DNA since the mutant T790M allele is heterozygous. As such, the representative WT allele fragment length distribution from tumor patients included WT alleles derived from tumor cells. This observation may explain, at least in part, why the differences in fragment length between the WT allele and the mutant T790M allele presented in Fig 4H were less pronounced than differences observed between the WT allele from healthy controls and the mutant T790M allele shown in Fig 4F.

### Cell-Free DNA Fraction Selection for Mutant Allele Enrichment in Human Lung Cancer

**Fig 5:**
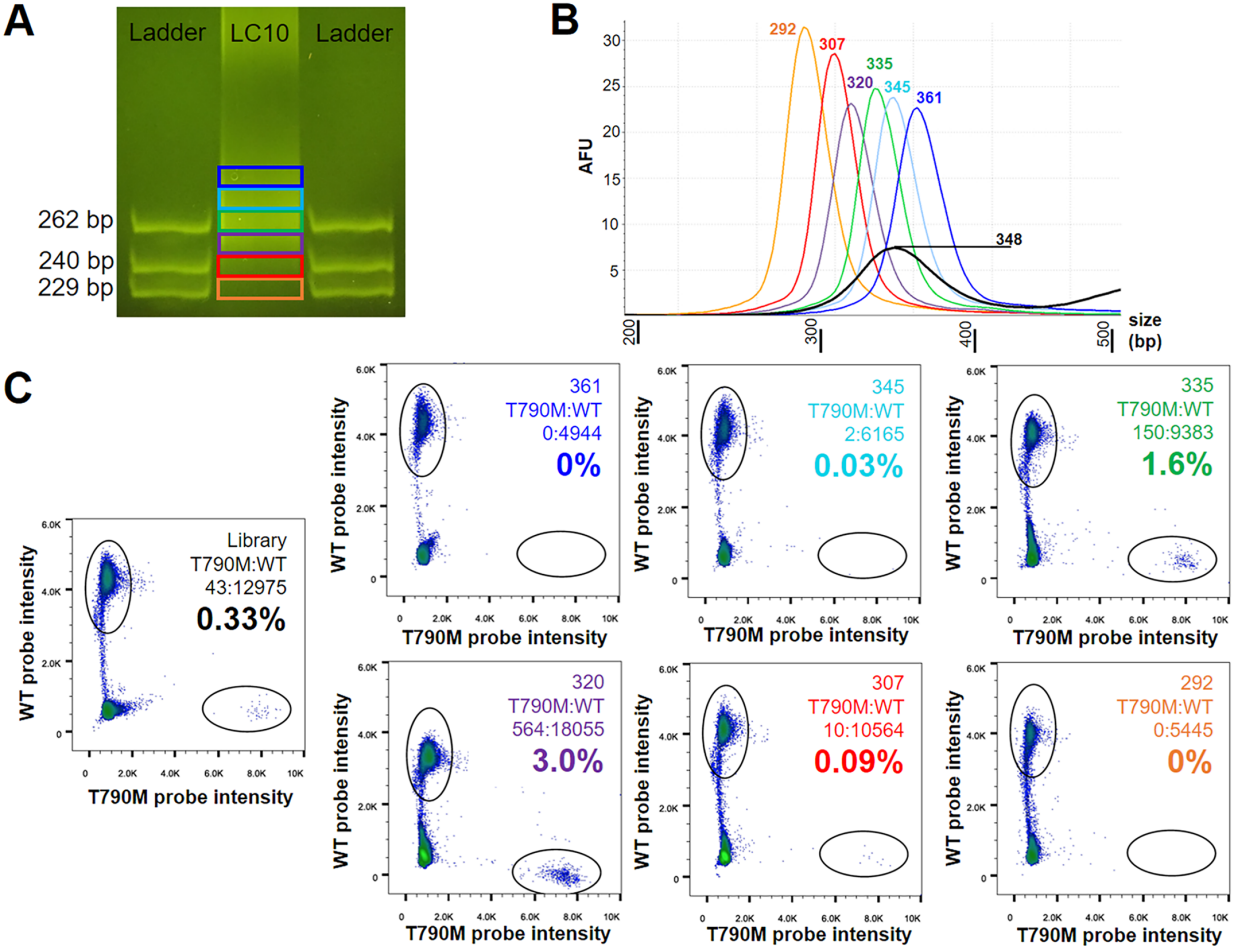
Extraction of cell-free DNA fractions for evaluating mutant allele frequency within specific fragment lengths. In **A**, an image of an 8% polyacrylamide gel loaded with a truncated library prepared from the cell-free DNA of a lung cancer patient (LC10, middle column). On either side is a custom-designed ladder made from phage lambda containing double-stranded DNA of 229, 240, and 262 bp in length. Six adjacent samples were excised from the gel corresponding to the colored boxes. In **B**, densitometry of the full-length libraries made from each fraction and the original library are shown. Colors of each curve and peak correspond to the colors in **A** (the library is shown in black). In **C**, the mutant allele frequency as determined by digital droplet PCR is shown for the library and each fraction. Colors for mutant allele frequency (%) correspond to the colors in **A** and **B**. Note that the purple fraction (peak fragment length of 320 bp) represented the largest increase (9.1-fold) in mutant allele frequency compared to the library (peak fragment length of 348 bp). Fractions containing longer fragment lengths than the library (e.g., blue fraction: peak fragment lengths of 361 bp) demonstrated a reduction in mutant allele frequency.

We next set out to determine whether selection for shorter fragment lengths could be used to enrich for ctDNA fragments against the large background of cell-free DNA derived from normal cells. Cell-free DNA sequencing libraries from four lung cancer patients (LC1, LC3, LC4, and LC10) with *EGFR* T790M mutations and one healthy control (C5) were selected for serial fraction collection. By sequencing, LC1 and LC3 had *EGFR* T790M mutant allele frequencies of 1.2% and 6.6%, respectively, and evidence of overall shorter cell-free DNA fragments compared to healthy controls (Fig 4B and S5B Fig, respectively). LC4 and LC10 had *EGFR* T790M mutant allele frequencies of 2.3% and 1.7%, respectively, and a similar size distribution of cell-free DNA fragments compared to healthy controls (S5G Fig and Fig 4C, respectively). None of the samples had an *EGFR* amplification present (S6 Fig). For each sample, 1 μg of sequencing library was loaded onto an 8% native polyacrylamide gel and six adjacent gel fragments were collected (S8 Fig). Extracted DNA (5–10 ng) from each gel fragment was then amplified using the full-length adapter primer and the *EGFR* T790M mutant allele frequency was determined via digital droplet PCR (Fig 5).

**Fig 6:**
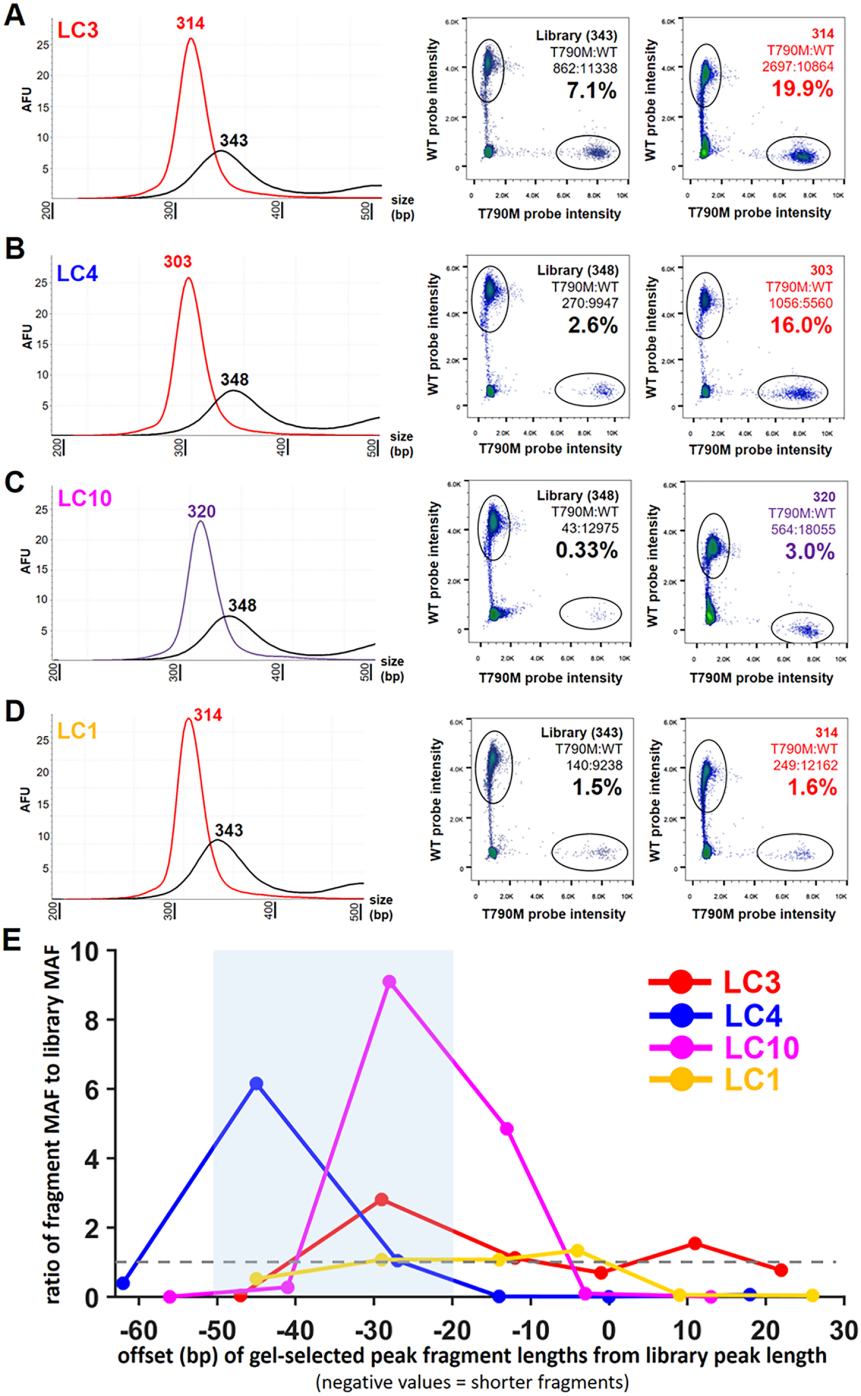
Selection of shorter cell-free DNA fragments enriched for ctDNA. In **A-D**, cell-free DNA fragment size distribution by densitometry and the corresponding digital droplet PCR results for mutant allele frequency are shown for each tumor patient. In **A-C**, the first column identifies the fragment size distribution for the fraction associated with the largest increase in mutant allele frequency (red or purple curve) along with the distribution of the corresponding library (black curve). In **D**, the first column shows a similar fraction for LC1 as presented in **A-C**. In **A-D**, the color of each curve matches the gel location as depicted in S8 Fig. In **A-D**, the middle and last columns report the digital droplet PCR results for mutant allele frequency (%) in the library and the gel fraction, respectively. In **E**, the ratio of the mutant allele frequency in each fraction to the MAF in the library was plotted for each tumor patient. The dashed gray line represents a ratio of 1 (i.e., no increase or decrease in MAF). To account for variability during gel fraction excision between samples, the *x*-axis location for plotting values associated with each gel fraction was determined via densitometry by subtracting the peak fragment length for each library from the peak fragment length for each fraction. Negative values correspond to shorter fragments and positive values correspond to longer fragments. The blue shaded box identifies the region where increase in the mutant allele frequency was the greatest across all samples.

Compared to the mutant allele frequency in the library, three samples (LC1, LC4, and LC10) demonstrated a 2.5-fold to 9.1-fold increase in the mutant allele frequency in a subset of fractions that contained a shorter distribution of cell-free DNA fragments relative to the peak fragment length in the library (Fig 6 and S9–S11 Figs). The fraction associated with the greatest increase in mutant allele frequency for each tumor patient is identified in Fig 6A–6C. In one sample (LC1), the mutant allele frequency did not increase in any fraction relative to the mutant allele frequency in the library (S12 Fig). However, a decrease in the mutant allele frequency was observed in fractions containing longer fragments, while fractions with shorter fragments contained a relatively consistent mutant allele frequency (Fig 6D and S12 Fig). Enrichment for the mutant allele was greatest in fractions that were centered approximately 20–50 bp shorter than the peak fragment length associated with each corresponding library (Fig 6E). The increase in mutant allele frequency was greatest in LC10 (Fig 5 and S11 Fig) and LC4 (S10 Fig), which were the two tumor patients with a similar fragment size distribution profile as that seen in the healthy controls (Fig 4C and S5G Fig, respectively). This finding suggests that the fractional selection of shorter cell-free DNA fragment lengths may improve mutant allele sensitivity when ctDNA is not the predominant component of cell-free DNA. Also notable is that the percentage of mutant allele detected in a sample low in ctDNA prior to enrichment may not represent the true allele frequency present in the tumor due to dilution by normal cell-free DNA. In LC1 (S12 Fig) and LC3 (S9 Fig), the tumor patients with evidence of overall shorter cell-free DNA fragments compared to healthy controls, the increase in mutant allele frequency in fractions 20–50 bp shorter than the peak fragment length associated with each library was not as substantial; however, selecting these fractions also did not diminish the mutant allele frequency (Fig 6E). In contrast, the selection of fractions longer than the library’s peak fragment length substantially reduced the mutant allele frequency in three of the tumor samples (LC1, LC4, and LC10; Fig 6E and S10–S12 Figs). Similarly, the selection of fractions containing cell-free DNA fragments >50 bp shorter than the library’s peak fragment length reduced the mutant allele frequency in all of the samples (Fig 6E). This latter observation may be a consequence of recovery during library preparation as discussed earlier (Fig 3D) [22]. Regardless, these observations provide compelling evidence that the fragment length of ctDNA is shorter than cell-free DNA from healthy cells and selection of shorter cell-free DNA fragments may improve mutant allele frequency. Of note, the *EGFR* T790M mutant allele was not present above the noise level associated with digital droplet PCR in the fractions obtained from the control sample (S13 Fig).

## Discussion

Our broad observation that the fragment length of ctDNA differs from cell-free DNA is supported by earlier reports that utilized amplicons of varying length to identify large categorical size differences between ctDNA associated with colorectal cancer and cell-free DNA from healthy controls [16, 24]. In addition, deep sequencing has been previously used to identify ctDNA shortening in hepatocellular carcinomas with specific aneuplodies [17]. However, this latter study also identified fragment lengths larger than healthy controls associated with low ctDNA concentrations in patients with hepatocellular carcinoma which is difficult to reconcile [17]. The collective findings described in our study builds upon these previous works by utilizing massively parallel sequencing to define distinct differences in fragment length between ctDNA and cell-free DNA. Specifically, animal models of GBM and hepatocellular carcinoma found that the most common fragment lengths of ctDNA were 134 and 144 bp, which was in contrast to the most common 167 bp fragment length present in normal cell-free DNA. These findings replicated in human patients with melanoma. Moreover, selection of cell-free DNA fractions containing shorter fragment lengths substantially increased mutant allele frequency in human lung cancer patients, particularly when the distribution of cell-free DNA fragment lengths in tumor patients was similar to the distribution seen in healthy controls. As such, the findings described herein provide strong evidence that a more general process that shortens ctDNA fragment length relative to normal cell-free DNA from healthy cells is present and is independent of copy number alterations.

The overall distribution of fragment lengths identified for ctDNA and cell-free DNA in our study was consistent with cellular apoptosis rather than necrosis [25]. In addition, the observed ~10 bp periodicity has been well-described in association with nuclease-cleaved nucleosome activity [26]. However, the etiology of the shorter fragment length associated with ctDNA remains unclear. Lo et al. previously reported similar findings from maternal serum with regards to fragment length differences between fetal cell-free DNA and maternal cell-free DNA [27]. Differences in cell-free DNA fragment lengths between donor-derived and host cell-free DNA in organ transplant patients has also been observed [28]. The extent of cell-free DNA shortening across disparate tissue contexts, in health and disease, suggests that tissue-specific processes may contribute to certain cell-free DNA fragment length sub-populations. One plausible hypothesis is that tissue-specific differences in nucleosome wrapping [29] result in fragment lengths that differ between hematopoietic cells (which contribute the majority of the plasma cell-free DNA) and other tissues of origin. Understanding the specific mechanism behind this phenomenon may prove valuable in oncology. Regardless of etiology, enriching for a specific subset of cell-free DNA fragment lengths may improve detection of ctDNA associated with non-metastatic solid tumors. More sensitive detection of mutations present in ctDNA may lead to non-invasive diagnosis of malignancy, improved detection of tumor recurrence, and better monitoring of response to therapy.

A limitation of this study was that very short rat cell-free DNA fragments (<100 bp) were detected in the GBM4_1_ animal (Fig 1F, red line) and very short human ctDNA fragments (<100 bp) were detected in the Control_1_ animal (S1D Fig, blue line) that were not present in the other animals. In the former, these very small fragments created a unique bimodal distribution of normal rat cell-free DNA. In the latter, these fragments were associated with an increased proportion of human ctDNA compared to other control animals (Fig 1C). As such, it was unclear if low levels (<0.01%) of ctDNA in tumor-bearing animals were a true signal or noise. Earlier use of a xenograft model for detection of ctDNA via PCR found a very high species sensitivity and specificity [24]. Although the very short fragments identified in our study were most likely secondary to contamination or sample handling, future xenograft-based studies utilizing species specific genomes obtained from massively parallel sequencing to separate ctDNA from cell-free DNA would benefit from determination of sensitivity and specificity. A second limitation is the accuracy of densitometry measurements (S14 Fig). Although densitometry tended to preserve relative differences between samples, we found that estimation of true fragment length was often over-estimated. As such, sequencing results may provide a more accurate measure of fragment length assuming sufficient reads of different sized inserts are available to reduce size profile noise.

## Materials and Methods

### Ethics Statement

All human subject research was approved by the University of Utah Institutional Review Board prior to study initiation. Written informed consent was obtained for samples from melanoma patients and healthy controls according to IRB approved studies 10924 and 7740. Informed consent was not obtained for the lung cancer samples as specimens were obtained from residual clinical samples scheduled for disposal and after de-identification according to IRB approved study 7275. Adult male RNU rats were used in this study. All procedures were approved by the University of Washington Internal Animal Care and Use Committee prior to study initiation. For surgery, rats were anesthetized with ketamine and xylazine administered IP. For imaging, rats were anesthetized with isoflurane mixed with oxygen. Rats were euthanized with Beuthanasia-D administered IP.

### Cell Culture

Established human GBM stem-like cell lines (GBM4, GBM8) [18, 19] were maintained in serum-free Neurobasal medium (ThermoFisher Scientific) with 2 mM glutamine, 5 μg/mL heparin, 100 U/ml penicillin-streptomycin, N2 at 0.5X, B27 supplement minus vitamin A at 0.5X, and bi-weekly pulsing of FGF and EGF (20 ng/mL each). Human hepatocellular carcinoma cells (Hep G2; ATCC) were maintained in Williams’ medium E with glutamine and 10% fetal bovine serum. All cells were maintained in a humidified incubator at 37°C in 5% CO_2_.

For implantation, single cell suspensions of GBM4 and GBM8 were achieved using heparin-EDTA and trituration followed by spin and wash ×2 with Neurobasal medium, then spin and resuspend in Neurobasal medium with DNase (4k U/ml), trituration, and incubation ×5 minutes at room temperature. Cells were then washed ×2 with Neurobasal medium to remove DNase and resuspended in Neurobasal medium for cell counting. Cells were counted with a hemacytometer after suspension in Trypan blue. For implantation, 1×10^6^ cells were resuspended in 10 μL of Neurobasal medium. Hep G2 cells were harvested by heparin-EDTA, counted using a hemocytometer after suspension in Trypan blue. For implantation, 5×10^6^ cells were resuspended in 100 μL of Williams’ medium E.

### Animal Procedures

Adult male RNU rats (Charles River Laboratories, Wilmington, MA) were used in this study. All procedures were approved by the University of Washington Internal Animal Care and Use Committee prior to study initiation.

Rats were anesthetized with 60 mg/kg ketamine and 5 mg/kg xylazine administered IP. For intracranial inoculation (GBM4 and GBM8), the head was immobilized in a stereotactic head set with ear bars and a teeth bar. The skull was exposed by a 2 cm midline incision, and a burr hole was created on the right side 1 mm anterior and 2 mm lateral to the bregma. A microsyringe (Hamilton, Reno, NV) was used to inject the 10 μL aliquot of 10^6^ cells into the frontal lobe at a depth of 5 mm from the skull surface over a period of 5 minutes. The needle was kept in place 2 minutes after injection to prevent backflow prior to removal. The burr hole was filled with bone wax (Ethicon, Somerville, NJ). The skin was closed with surgical staples that were removed prior to MR imaging. For flank injections (Hep G2), a 22-gauge needle attached to a TB syringe was used to inject the cells subcutaneously into the right flank.

After the final imaging time point, the rats were anesthetized with Beuthanasia-D (2 mL/kg). A midline abdominal incision followed by thoracotomy was made to access the left ventricle of the heart. A 22-gauge needle attached to a syringe containing heparin was used to remove as much blood as possible (6–10 mL). Subsequently, 4% paraformaldehyde (PFA) was injected into the left ventricle (total volume 150 mL) as the right atrium was opened. Brains were subsequently removed intact, held in 4% PFA×24 hours under gentle agitation, and then maintained in PBS.

### Histology

After fixation, brains were sectioned to correspond with the anatomic coronal plane. Brains were subsequently embedded in paraffin and sections (5 μm thick) were stained with hematoxylin-eosin. Stained slides were scanned using an Olympus VS110 virtual microscopy system (Olympus, Center Valley, PA) for display on NDP.view (v2.3.1).

### Animal Imaging

Rats were imaged on a 3.0 T Philips Achieva whole-body MRI scanner (Philips Medical Systems, Best, Netherlands) using a dual coil approach. A quadrature transmit/receive head coil (Philips Medical Systems) was utilized for RF transmission, and an in-house-built combined solenoid-surface coil [30] dedicated to high spatial resolution whole-brain rat imaging was used for RF reception. After induction in an anesthesia chamber with 5% isoflurane mixed with oxygen, the rats were positioned within the dual coils and maintained on 2% isoflurane mixed with oxygen via nose cone inhalation. Total scan time for all images was < 1 hour.

### Bound-Pool Fraction Map Acquisition

Images necessary for construction of bound-pool fraction maps in the rat brain were acquired as previously described [20]. Briefly, *Z*-spectra data points were acquired for each rat using a 3D MT-prepared spoiled gradient echo (GRE) sequence with TR/TE = 42/4.6 ms, excitation flip angle α = 10°, NEX = 1, and three offset frequencies (Δ = 4, 8, and 96 kHz) of the off-resonance saturation pulse (effective flip angle = 950°). Complementary *R*_1_ maps necessary for parameter fitting were obtained using the variable flip angle method [21] with a 3D spoiled GRE sequence (TR/TE = 20/2.3 ms, α = 4 (NEX = 3), 10 (NEX = 1), 20 (NEX = 2), and 30° (NEX = 3)). All *Z*-spectral and VFA images were acquired with a FOV = 29×29×19.8 mm^3^, matrix = 97×97×66, acquisition resolution = 0.3×0.3×0.3 mm^3^ (zero-interpolated to 0.15×0.15×0.15 mm^3^), and full-Fourier acquisition. Whole-brain 3D *B*_0_ and *B*_1_ maps were acquired to correct for field heterogeneities. For *B*_0_ mapping, the GRE-based dual-TE phase-difference method [31] was used with TR/TE_1_/TE_2_ = 20/4.7/5.7 ms and α = 10°. *B*_1_ maps were obtained using the actual flip angle imaging method [32] and the following sequence parameters: TR_1_/TR_2_/TE = 25/125/6.6 ms and α = 60°. 3D *B*_0_ and *B*_1_ maps were acquired with a FOV = 29×29×19.8, matrix = 64×64×33, acquisition resolution = 0.45×0.45×0.6 mm^3^ (zero-interpolated to 0.15×0.15×0.15 mm^3^), and NEX = 1. All images were acquired in the coronal plane.

### Contrast-Enhanced MRI

For administration of gadolinium (gadopentetate dimeglumine, Bayer HealthCare; 0.5 M/L) a 22 Gauge angiocatheter (Becton-Dickinson, Sandy, Utah) was inserted into the rat tail vein. The catheter was attached to a small bore bifurcated extension (Smiths Medical, Dublin, OH) containing a dilution of gadolinium in one arm and a normal saline flush in the other. The catheter setup was maintained with a saline lock until immediately prior to imaging. At time of injection, 0.2 mmol/kg (0.167 M/L) of gadolinium was manually injected at 50 μL/s followed by a 250 μL flush of normal saline at 50 μL/s. Complementary pre-contrast *R*_1_ maps necessary for parameter fitting and post-contrast *R*_1_ maps obtained 5 minutes after contrast injection were acquired using the variable flip angle method [21] with a 3D SPGR sequence (TR/TE = 4.6/20 ms, α = 4 (NEX = 3), 10 (NEX = 1), 20 (NEX = 2), and 30° (NEX = 3)) and FOV = 24×24×8.25 mm^3^, matrix = 64×64×5.5 for an acquisition resolution of 0.38×0.38×1.5 mm^3^ (zero-interpolated to 0.19×0.19×0.75 mm^3^). Pre-contrast *B*_1_ maps using the actual flip angle imaging method [32] were acquired with the following parameters: TR_1_/TR_2_/TE = 25/125/6.7 ms and α = 60°. 3D *B*_1_ maps were acquired with a NEX = 1 and an identical resolution as the variable flip angle data points. All images were acquired in the axial plane.

### Image Processing

Fast bound-pool fraction parametric maps (*f* maps) were constructed consistent with a previously described methodology [20] for single parameter determination of *f*. Briefly, *R*_1_ maps were used to define *R*_1_^F^ and reconstructed from VFA data using a linear fit to the signal intensities (*S*) transformed into the coordinates [*S*(α) / sin α, *S*(α) / tan α][21] after voxel-based *B*_1_ corrections were applied to α. In the MT data, the Δ = 96 kHz *Z*-spectra images were used to normalize the Δ = 4 and 8 kHz data points and voxel-based *B*_0_ and *B*_1_ corrections were applied to Δ and α, respectively, during voxel-based fitting for *f*. The parameters *k*, *T*_2_^F^*R*_1_^F^, and *T*_2_^B^ were constrained to 29 x *f*/(1-*f*) s^-1^, 0.030, 10.7 μs, respectively, as previously determined [20]. *R*_1_^B^, the longitudinal relaxation of the bound-pool, was set to a fixed value of 1 s^-1^ by convention [33–35].

Pre- and post-contrast *R*_1_ maps were similarly constructed from the respective VFA data that was acquired in the axial plane. Corresponding pre-contrast *B*_1_ maps were similarly applied for correction of α during fitting of both pre- and post-contrast *R*_1_ maps. Image processing dedicated to whole-brain voxel-based determination of *f* maps and *R*_1_ maps was performed using in-house written Matlab (The Mathworks, Natick, MA) and C/C++ language software.

### Rat Samples: Plasma Collection, Storage, and Cell-Free DNA Isolation

Whole blood acquired from each animal was centrifuged at 1,600 g ×10 minutes at 4°C. The plasma layer was removed and centrifuged at 16,000 g ×10 minutes at 4°C. The buffy coat was then collected and stored at -80°C. After centrifugation, plasma was removed excepting a residual amount near the bottom that may have been in contact with any debris and stored at -80°C. Both plasma samples and the buffy coat were stored at -80°C <1 hour from time of collection from the animal.

DNA was isolated from buffy coat cell pellets using the Qiagen DNeasy Blood and Tissue kit. Shotgun sequencing libraries were constructed with 50 nanograms of gDNA from each animal using the Nextera DNA library prep kit (Illumina). Following the manufacturer’s direction, sample index sequences were added during the PCR step to allow libraries to be pooled for multiplexed sequencing on a single lane.

Cell-free DNA was extracted from rat plasma using the QIAamp Circulating Nucleic Acid kit. DNA yield was measured with a Qubit dsDNA HS assay (Invitrogen) and 1–10 ng of cell-free DNA was used as input for library construction with the Thruplex-LC kit (Rubicon Genomics). For samples with low input concentration (<100 pg/ul), cell-free DNA was first concentrated across Zymo Clean-Concentrate-5 column (Zymo Research).

### Rat Samples: NGS Library Preparation, Sequencing, and Bioinformatics

During library construction, enrichment PCR was performed using a BioRad MiniOpticon real-time thermocycler, with SYBR Green I dye (Invitrogen) added to each reaction at a final concentration of 0.25X. Reactions were individually removed upon entering log-phase amplification as indicated by SYBR signal (7–17 cycles).

Libraries were normalized to 2 nM each and pooled for paired-end 101-bp sequencing across four lanes on an Illumina Hiseq 2000 instrument. A 9-bp index read was also collected and used to demultiplex reads according to input sample, requiring fewer than 2 mismatches to the known indices.

For each buffy coat and cell-free DNA library, adapter sequences were trimmed and paired end reads were mapped to human and rat reference assemblies (hg19 and rn5, respectively) using bwa [36]. For each read pair, the species origin (rat or human) was then determined using the mapping status against both references. Only reads that could be unambiguously mapped to one or the other species were included: reads with low mapping quality score (<30) in both species’ references were discarded, as were reads of comparable mapping quality to both references (absolute difference in map quality scores <20). Tumor DNA abundance in each cell-free DNA and buffy coat fraction was then computed as (# human read pairs) / (# human read pairs + #rat read pairs). Fragment length were then takes as the absolute distances between the outermost bases of each pair of forward and reverse ends.

As a quality control check, an aliquot of each xenografted cell line at the time of implantation was genotyped across a panel of 96 human polymorphisms using a custom BeadArray assay performed by the Northwest Genomics Center. Cell lines with identical genotype calls in ≥ 95 of 96 markers were considered to be identical in origin, whereas all other pairs of cell lines shared genotypes at many fewer markers (34–45; S15 Fig).

### Human Samples: Plasma Collection, Storage, and Cell-Free DNA Isolation

All procedures were approved by the University of Utah Internal Review Board prior to study initiation. Blood samples were collected in Streck BCT tubes, stored at 4°C, and processed within 24 hours of collection. Plasma was separated by centrifugation for 10 minutes at 1900g and aspiration to a new tube. Plasma was further centrifuged for 16,000g x 10 minutes to remove any cellular debris, and resulting supernatant was stored at –20°C until cell-free DNA isolation. Custom kits that combined Qiagen lysis and binding buffer with Zymo silica-based columns were assembled to reduce expense during isolation of cell-free DNA. Cell-free DNA was prepared from 8 mL of plasma by adding 800 μL of Proteinase K (20 mg/mL) and 6.4 mL Buffer ACL (Qiagen) followed by incubation at 60°C x 30 minutes. Next, 14.4 mL of buffer ACB (Qiagen) was added to the lysate and incubated on ice for 5 minutes. DNA was isolated from the lysate with Zymo DNA Clean and Concentrator 100 kit according to the manufacturer’s instructions and eluted in 150 μL. A final purification step was performed using two volumes of Ampure XP magnetic beads followed by elution in 25–30 μL 10mM Tris (pH 8.0).

### Human Samples: NGS Library Preparation, Sequencing, and Bioinformatics

#### Melanoma Samples

Libraries for Illumina high throughput sequencing were prepared using the KAPA biosystems Hyper Prep Kit with either 40 ng (pooled normal) or 100 ng (melanoma) of input cell-free DNA according to the manufacturer’s instructions. Cell-free DNA libraries were enriched for regions of interest using a custom designed IDT Xgen capture probe set containing full exonic coverage of the following genes: *AKT1*, *ALK*, *BRAF*, *EGFR*, *ERBB2*, *ESR1*, *KIT*, *KRAS*, *MAP2K1*, *MET*, *MTOR*, *NRAS*, *PDGFRA*, *PIK3CA*, *PTCH1*, *PTEN*, *SMO*, and *TP53*. Paired-end sequencing of libraries was performed on an Illumina MiSeq. FASTQ files were aligned to the HG19 assembly of the human genome using bwa mem (v. 0.7.9a-r786) and insert size metrics for each sample were assessed using Picard tools (v. 1.107). Tumor fraction as a function of insert size was analyzed using the Pysam samtools API.

#### Lung Cancer Samples

Libraries for Illumina high throughput sequencing were prepared using the KAPA biosystems Hyper Prep Kit with 10 ng input cell-free DNA according to the manufacturer’s instructions using truncated duplex molecular barcode adapters [37]. A 100 ng aliquot of the library was further amplified using the Kapa HiFi 2x master mix with the full length adapter primer containing the sample specific index. Cell-free DNA libraries were enriched for regions of interest using a custom designed IDT Xgen capture probe set containing full exonic or hotspot coverage of the following genes: *AKT1*, *ALK*, *BRAF*, *CTNNB*, *DDR2*, *EGFR*, *ERBB2*, *KIT*, *KRAS*, *MAP2K1*, *MET*, *MTOR*, *NRAS*, *PIK3CA*, *PTEN*, and *TP53*. Paired-end sequencing of libraries was performed on an Illumina HiSeq 2500. Reads in FASTQ files were collapsed into unique observations based on molecular barcodes and alignment information. Reads were aligned to the GRCh37 reference genome using bwa mem (v. 0.7.12a-r1044). Fragment length was derived from paired-end alignment information according to SAM format [38]. Overlapping read pairs were treated as single observations, and barcodes observed only once were omitted from analysis due to their relatively higher error rates. Copy number alterations were identified with the mean read depth of unique observations for each gene.

### Human Samples: Fragment Selection and Digital Droplet PCR

#### Polyacrylamide Gel Excision and Extraction

Selected libraries made with the truncated barcoded adapters described above were loaded (1 μg) on an 8% native polyacrylamide gel. A custom-made ladder of double-stranded DNA fragment was loaded into the wells adjacent to the sample. The ladder was constructed from lambda phage using Hot Start Taq DNA polymerase (Roche) and the following primer pairs:

262 bp: 5’–CATCTGCTTCTGCTTTCGCC–3’ and 3’–CTGGGTATTTCCCGGCCTTT–5’

240 bp: 5’–GGAACCCACCGAGTGAAAGT–3’ and 3’–ACTCTTTCCATGCCGCTTCA–5’

229 bp: 5’–GATGGCTCGCCAGTTCCATA–3’ and 3’–ACCAATATCCAGCACCGCAT–5’

Ladder lengths were selected to generally reflect the size of normal and tumor-derived cell-free DNA fragments observed by sequencing after the addition of the truncated adapters (~99 bp). The ladder contained 75 ng of each of the three fragment lengths. Six consecutive fragments were selected from the gel for DNA extraction after the gel was incubated in TBE with SYBER safe (Thermofisher) per the manufacturer’s instructions at RT x 30 minutes on a gentle shaker. Individual gel pieces were disrupted with Gel Breaker Tubes (IST Engineering Inc., Milpitas, CA), suspended in diffusion buffer (0.5 M ammonium acetate; 10 mM magnesium acetate; 1 mM EDTA, pH 8.0; 0.1% SDS), placed in a heating block at 50°C for 1 hour and then placed on a shaker at room temperature overnight. Gel pieces were removed by passing the sample through a 5 μM filter tube (IST Engineering Inc.). Three volumes of QG buffer (Qiagen) were then added to the sample which was subsequently applied to a QIAquick Spin Column (Qiagen). DNA was extracted from the column following the manufacturer’s protocol for the QIAquick PCR Purification Kit (Qiagen) and eluted in 30 μL. A 5–10 ng aliquot of each fraction and 10 ng of the original library was then amplified using the Kapa HiFi 2x master mix with the full length adapter primer containing a sample specific index. Samples were purified using a matched volume (1x) of Ampure XP magnetic beads followed by elution in 25 μL ATE. A TapeStation 2200 (Agilent Technologies) was used to evaluate fragment distribution.

#### Digital Droplet PCR

*EGFR* T790M mutant allele frequencies were determined by picoliter digital droplet PCR (RainDance Technologies) using a droplet size of 5 pL. PCR primers, hydrolysis probes, and amplification conditions were implemented exactly as described in the study by Milbury et al. [39]. Total PCR reaction volume prepared was 25 μL and contained ~100 ng of cell-free DNA. All primers and probes were synthesized by Integrated DNA Technologies. Droplet counts were determined using the RainDrop Analyst software. For each sample, the amplified library prior to size selection was used to define gates of *EGFR* wildtype and T790M droplet populations.

### Statistical Analysis

For continuous variables, the means and standard deviations (SDs) were calculated for each group. The student’s independent t-test assuming equal or unequal variance based on Levene’s test was used to compare mean values between tumor patients and healthy controls. Pearson’s *r* was used to identify correlations between continuous variables. Statistical analyses were performed with SPSS for Windows (Version 12.0, SPSS, Chicago, IL). Statistical significance was defined as *P* < 0.05.

## Supporting Information

S1 Fig Fragment length of cell-free DNA from control, GBM4, and GBM8 animals. In **A**, coronal bound-pool fraction maps (*f* maps) and pre- and post-contrast *R*_1_ maps with matched histology (**B**) and percent of human ctDNA detected in rat plasma (**C**, colored arrows identify results that correspond to images in A). GBM8_2_ was largely an invasive tumor (**A**) with minimal contrast enhancement (**B**) and good detection of ctDNA (**C**). In **D**, the percentage of rat cell-free DNA and human ctDNA according to fragment length is depicted for animals shown in (**A**) and for animal data not previously shown in Fig 1. In the control animals, GBM4_1_, and GBM4_2_ the ctDNA distribution (blue line) is erratic due to few observations.
(TIF)

S2 Fig Fragment length of cell-free DNA from control, GBM4, and GBM8 animals. In **A**, percent of human of human ctDNA detected in rat plasma for animals described in Fig 2, animals implanted with Hep G2 cells, and animals corresponding to coronal bound-pool fraction maps (*f* maps, colored arrows in A identify results that correspond to images in **B**) and pre- and post-contrast *R*_1_ maps (**B**) with matched histology (**C**). GBM4_5_ was a well circumscribed tumor that seemed to be dural-based as there was no evidence of intra-parenchymal tumor growth on histology (**C**; asterisk corresponds to 2x magnification of tissue that was loosely attached at location of asterisk). Despite strong contrast-enhancement and reasonable tumor size (**B**), detection of ctDNA was low (**A**, red arrow). GBM8_5_ was a relatively large tumor with modest contrast enhancement (**B**). However, detection of human ctDNA was only modestly elevated and the fragment length distribution was irregular with only very mild evidence of an increased fragment distribution in the 134 to 144 bp range (**D**). In **D**, fragment distribution for human ctDNA was largely erratic due to few observations.
(TIF)

S3 Fig In lung cancer patients, a correlation between fragment length size and concentration of cell-free DNA in plasma was not present. In **A**, plasma concentration of cell-free DNA from lung cancer patients was significantly higher compared to healthy controls, although substantial overlap between groups was present. In **B**, boxplots of the peak fragment length by densitometry for lung cancer patients and healthy controls found variability in both cohorts; however, the fragment length of tumor patients was significantly shorter compared to controls (*p* = 0.002). In **C**, peak fragment length and overall cell-free DNA concentration were not significantly associated in either the lung cancer patients (Pearson’s *r* = –0.20, *p* = 0.47) or the healthy controls (Pearson’s *r* = 0.19, *p* = 0.63; Fig 4C).
(TIF)

S4 Fig Limitations of analyzing fragment size distribution with densitometry using original cell-free DNA samples. Results from TapeStation analysis are shown for four control cell-free DNA samples (**A-D**) using the original cell-free DNA as input (left column) and the corresponding truncated adapter library as input (right column). In **A**, there is good identification of the upper and lower markers and a distinct peak for the cell-free DNA when using the original sample (left column). In **B** and **C**, the location of the upper marker (left column, red arrows) was ambiguous. Incorrect identification of the upper marker will substantially alter the fragment length of the peak associated with cell-free DNA. In **D**, the frequently low concentration of cell-free DNA in plasma from healthy controls led to a peak doublet (left column, red arrow) causing ambiguous determination of the actual peak fragment length. Utilizing the truncated adapter library enabled clear identification of the upper and lower marker and loading of an identical amount (2 ng/μL) of cell-free DNA for each sample (**A-D**, right column).
(TIF)

S5 Fig Fragment length determined by sequencing for each lung cancer patient compared to healthy controls. In **A-G**, the red line represents the fragment length distribution for a lung cancer patient, while the blue lines are the fragment length distribution for the five healthy controls. The plasma concentration of cell-free DNA and presence (+)/absence (-) of *EGFR* and *KRAS* amplifications are identified. In **H**, the fragment length distribution for only the healthy controls is shown along with the range of cell-free DNA plasma concentrations.
(TIF)

S6 Fig Heat map of mean read depth of unique observations for each of the 16 genes on the panel used to sequence the cell-free DNA from the five controls and seven lung cancer patients. LC5 demonstrated amplification of *EGFR* and *KRAS*. LC9 had amplification of *EGFR*. No additional amplifications were evident.
(TIF)

S7 Fig Fragment length determined by sequencing for the WT allele in each lung cancer patient compared to the healthy controls. In **A-G**, the red line represents the fragment length distribution of the WT allele in a lung cancer patient, while the blue lines are the fragment length distribution of the WT allele in the five healthy controls. The plasma concentration of cell-free DNA and presence (+)/absence (-) of *EGFR* and *KRAS* amplifications are identified for the tumor patients. In **H**, the fragment length distribution of the WT allele for only the healthy controls is shown along with the range of cell-free DNA plasma concentrations.
(TIF)

S8 Fig Representative images of the polyacrylamide gel showing the truncated adapter library and the ladder used to guide fraction selection. The ladder contained double-stranded DNA derived from phage lambda with lengths of 262 bp, 240 bp, and 229 bp. Using this ladder as a guide, six fractions were acquired from each library (far column).
(TIF)

S9 Fig Distribution of gel fractions and corresponding results from digital droplet PCR for LC3. In **A**, the gel image of the library (L) and six fractions (colored numbers correspond to gel locations in S8 Fig) after amplification using the full-length adapter primers. In **B**, the fragment size distribution of each fraction (blue, light blue, green, purple, yellow, red line) and the library (black line) are shown. The fragment length associated with the peak is identified for each sample in a corresponding color. In **C**, the mutant allele frequency for the library and each fraction via digital droplet PCR are identified. In **A-C**, all colors indicate corresponding samples and are consistent with the colors used in S8 Fig.
(TIF)

S10 Fig Distribution of gel fractions and corresponding results from digital droplet PCR for LC4. In **A**, the gel image of the library (L) and six fractions (colored numbers correspond to gel locations in S8 Fig) after amplification using the full-length adapter primers. In **B**, the fragment size distribution of each fraction (blue, light blue, green, purple, yellow, red line) and the library (black line) are shown. The fragment length associated with the peak is identified for each sample in a corresponding color. In **C**, the mutant allele frequency for the library and each fraction via digital droplet PCR are identified. In **A-C**, all colors indicate corresponding samples and are consistent with the colors used in S8 Fig.
(TIF)

S11 Fig Distribution of gel fractions and corresponding results from digital droplet PCR for LC10. In **A**, the gel image of the library (L) and six fractions (colored numbers correspond to gel locations in S8 Fig) after amplification using the full-length adapter primers. In **B**, the fragment size distribution of each fraction (blue, light blue, green, purple, yellow, red line) and the library (black line) are shown. The fragment length associated with the peak is identified for each sample in a corresponding color. In **C**, the mutant allele frequency for the library and each fraction via digital droplet PCR are identified. In **A-C**, all colors indicate corresponding samples and are consistent with the colors used in S8 Fig.
(TIF)

S12 Fig Distribution of gel fractions and corresponding results from digital droplet PCR for LC1. In **A**, the gel image of the library (L) and six fractions (colored numbers correspond to gel locations in S8 Fig) after amplification using the full-length adapter primers. In **B**, the fragment size distribution of each fraction (blue, light blue, green, purple, yellow, red line) and the library (black line) are shown. The fragment length associated with the peak is identified for each sample in a corresponding color. In **C**, the mutant allele frequency for the library and each fraction via digital droplet PCR are identified. In **A-C**, all colors indicate corresponding samples and are consistent with the colors used in S8 Fig.
(TIF)

S13 Fig Distribution of gel fractions and corresponding results from digital droplet PCR for the negative control (C5). In **A**, the gel image of the library (L) and six fractions (colored numbers correspond to gel locations in S8 Fig) after amplification using the full-length adapter primers. In **B**, the fragment size distribution of each fraction (blue, light blue, green, purple, yellow, red line) and the library (black line) are shown. The fragment length associated with the peak is identified for each sample in a corresponding color. In **C**, the mutant allele frequency for the library and each fraction via digital droplet PCR are identified. In **A-C**, all colors indicate corresponding samples and are consistent with the colors used in S8 Fig.
(TIF)

S14 Fig Densitometry profile of ladder used during fraction selection from polyacrylamide gels. The ladder was constructed from phage lambda double-stranded DNA consisting of three lengths: 229 bp (red), 240 bp (blue), and 262 bp (magenta). An image of the gel is shown in **A**. In **B**, peak fragment length as measured by densitometry is identified for each element of the ladder above the corresponding peak. Estimation of fragment length by densitometry was susceptible to an overestimation up to ~10 bp. Relative differences in the ladder were better preserved during polyacrylamide gel electrophoresis (S8 Fig).
(TIF)

S15 Fig Genotype using 96 human polymorphisms of cell lines (GBM4, GBM8, Hep G2) utilized in xenograft experiments. (TIF)
