# Supplementary figures and images for: Fragment Length of Circulating Tumor DNA
Source: PLoS Genet. 2016 Jul 18;12(7):e1006162. doi: 10.1371/journal.pgen.1006162 (PMC4948782; doi:10.1371/journal.pgen.1006162)

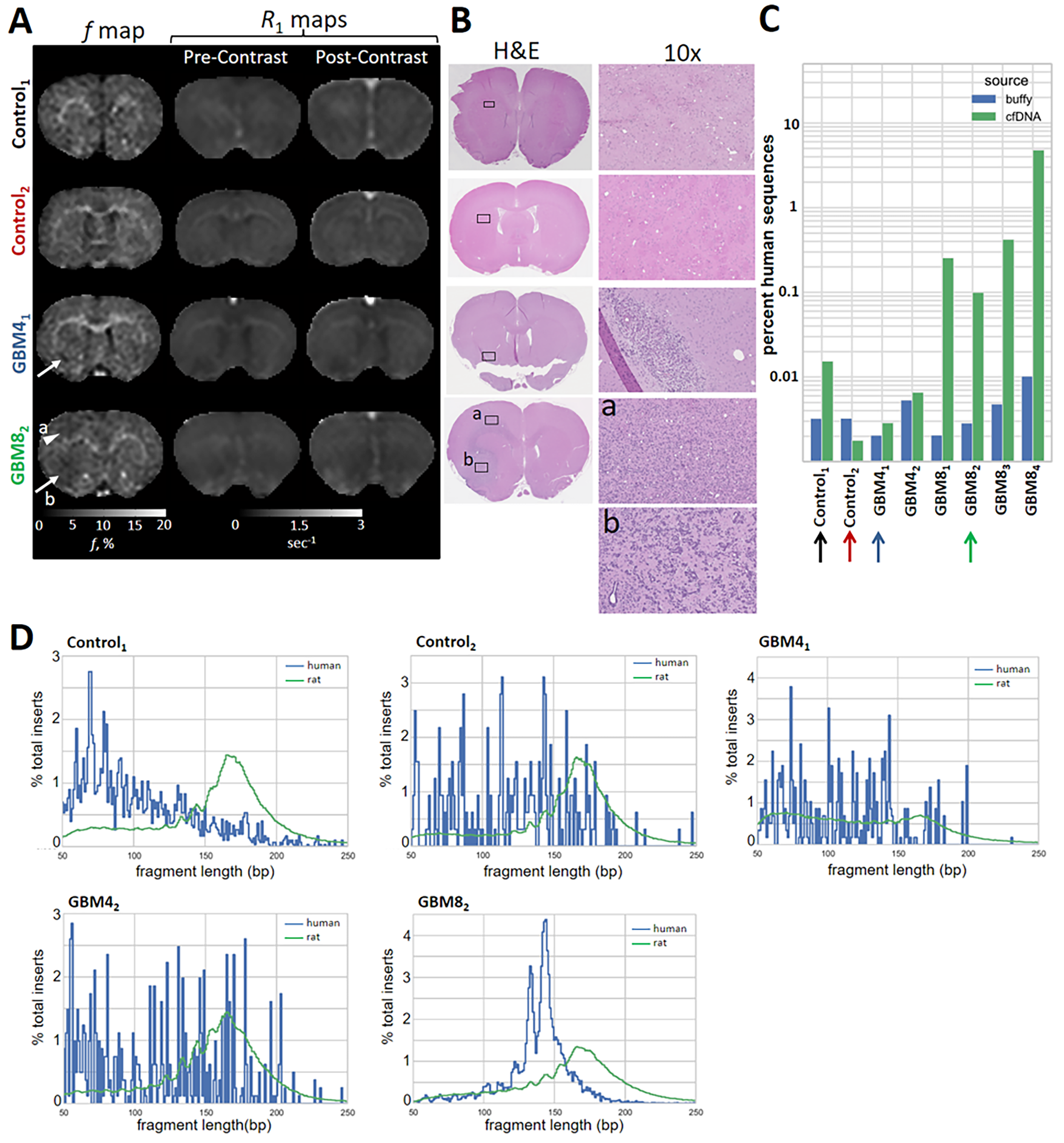

Supplement: S1 Fig — In A, coronal bound-pool fraction maps (f maps) and pre- and post-contrast R1 maps with matched histology (B) and percent of human ctDNA detected in rat plasma (C, colored arrows identify results that correspond to images in A). GBM82 was largely an invasive tumor (A) with minimal contrast enhancement (B) and good detection of ctDNA (C). In D, the percentage of rat cell-free DNA and human ctDNA according to fragment length is depicted for animals shown in (A) and for animal data not previously shown in Fig 1. In the control animals, GBM41, and GBM42 the ctDNA distribution (blue line) is erratic due to few observations. (TIF) [file pgen.1006162.s001.tif]

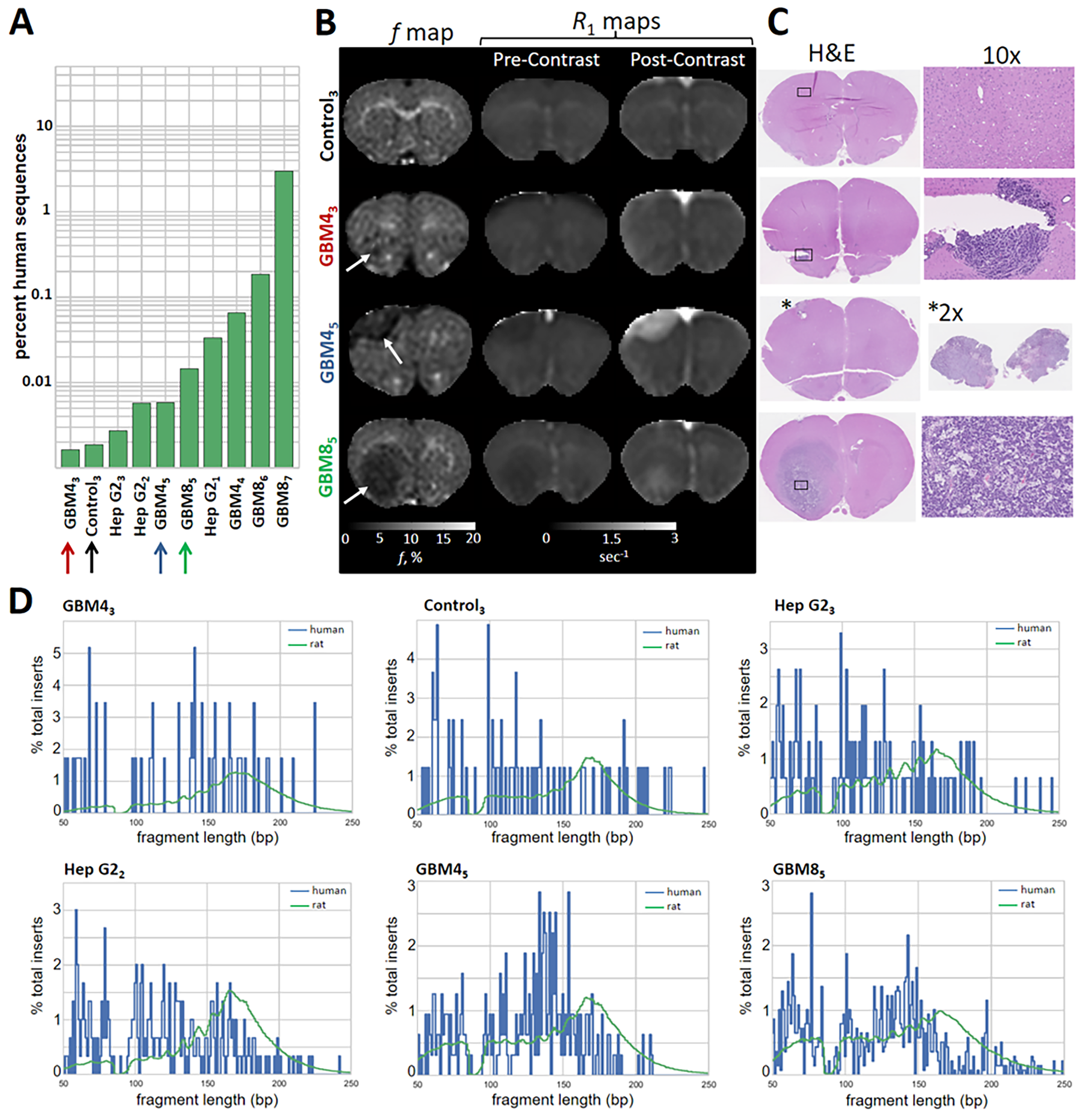

Supplement: S2 Fig — In A, percent of human of human ctDNA detected in rat plasma for animals described in Fig 2, animals implanted with Hep G2 cells, and animals corresponding to coronal bound-pool fraction maps (f maps, colored arrows in A identify results that correspond to images in B) and pre- and post-contrast R1 maps (B) with matched histology (C). GBM45 was a well circumscribed tumor that seemed to be dural-based as there was no evidence of intra-parenchymal tumor growth on histology (C; asterisk corresponds to 2x magnification of tissue that was loosely attached at location of asterisk). Despite strong contrast-enhancement and reasonable tumor size (B), detection of ctDNA was low (A, red arrow). GBM85 was a relatively large tumor with modest contrast enhancement (B). However, detection of human ctDNA was only modestly elevated and the fragment length distribution was irregular with only very mild evidence of an increased fragment distribution in the 134 to 144 bp range (D). In D, fragment distribution for human ctDNA was largely erratic due to few observations. (TIF) [file pgen.1006162.s002.tif]

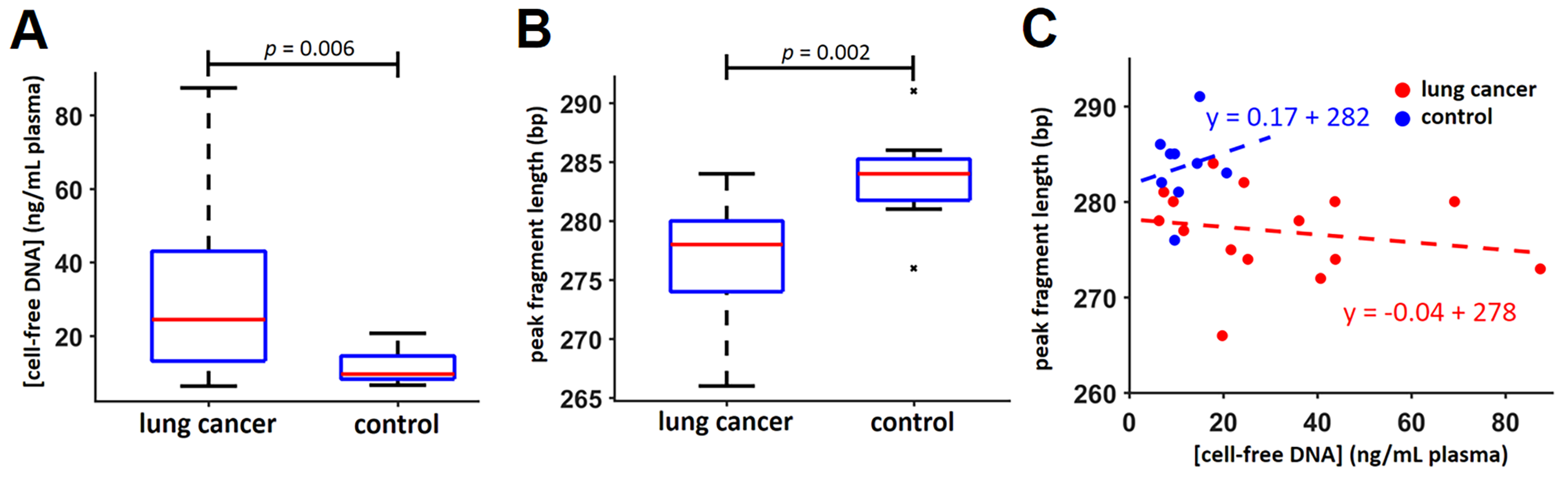

Supplement: S3 Fig — In A, plasma concentration of cell-free DNA from lung cancer patients was significantly higher compared to healthy controls, although substantial overlap between groups was present. In B, boxplots of the peak fragment length by densitometry for lung cancer patients and healthy controls found variability in both cohorts; however, the fragment length of tumor patients was significantly shorter compared to controls (p = 0.002). In C, peak fragment length and overall cell-free DNA concentration were not significantly associated in either the lung cancer patients (Pearson’s r = –0.20, p = 0.47) or the healthy controls (Pearson’s r = 0.19, p = 0.63; Fig 4C). (TIF) [file pgen.1006162.s003.tif]

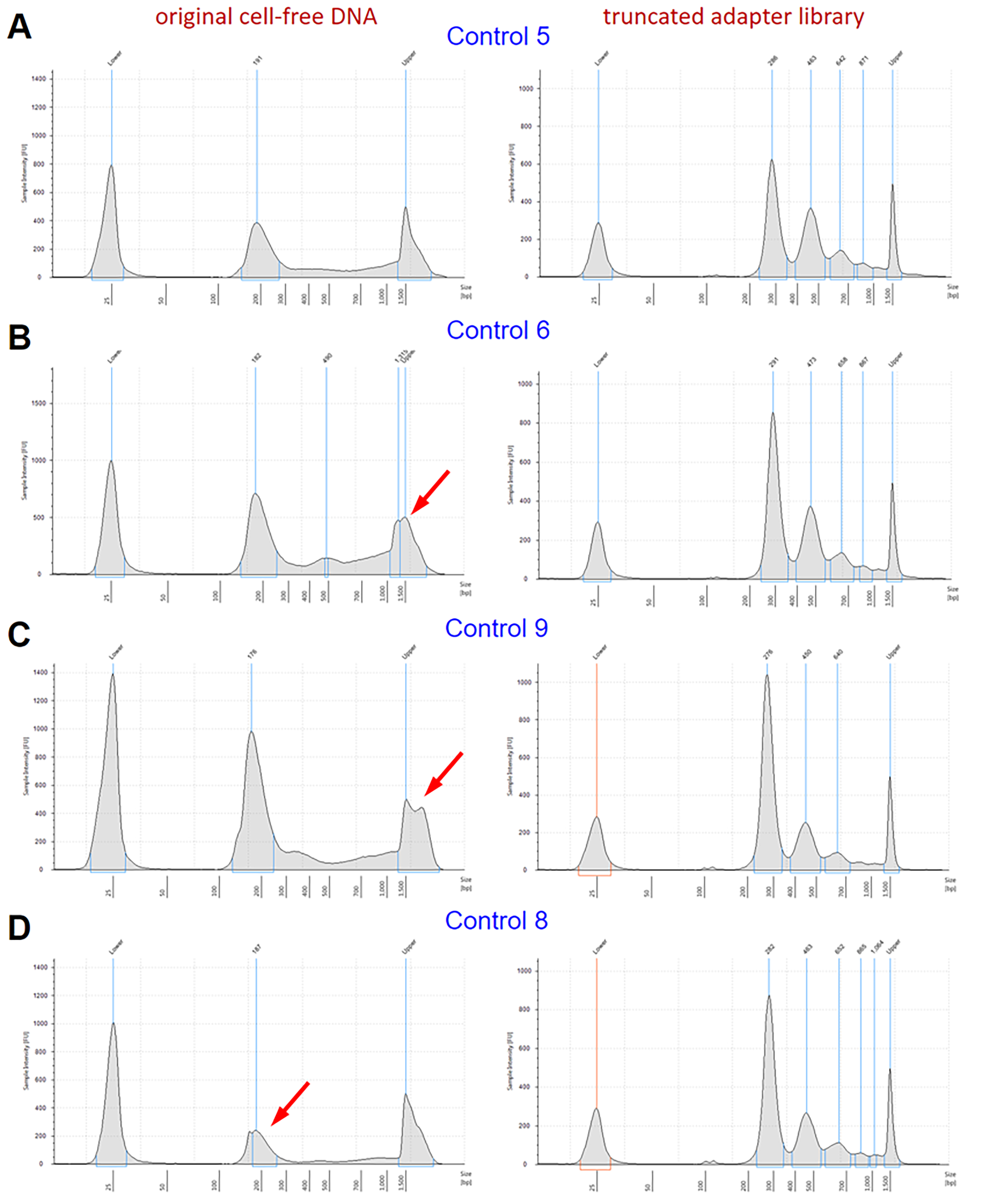

Supplement: S4 Fig — Results from TapeStation analysis are shown for four control cell-free DNA samples (A-D) using the original cell-free DNA as input (left column) and the corresponding truncated adapter library as input (right column). In A, there is good identification of the upper and lower markers and a distinct peak for the cell-free DNA when using the original sample (left column). In B and C, the location of the upper marker (left column, red arrows) was ambiguous. Incorrect identification of the upper marker will substantially alter the fragment length of the peak associated with cell-free DNA. In D, the frequently low concentration of cell-free DNA in plasma from healthy controls led to a peak doublet (left column, red arrow) causing ambiguous determination of the actual peak fragment length. Utilizing the truncated adapter library enabled clear identification of the upper and lower marker and loading of an identical amount (2 ng/μL) of cell-free DNA for each sample (A-D, right column). (TIF) [file pgen.1006162.s004.tif]

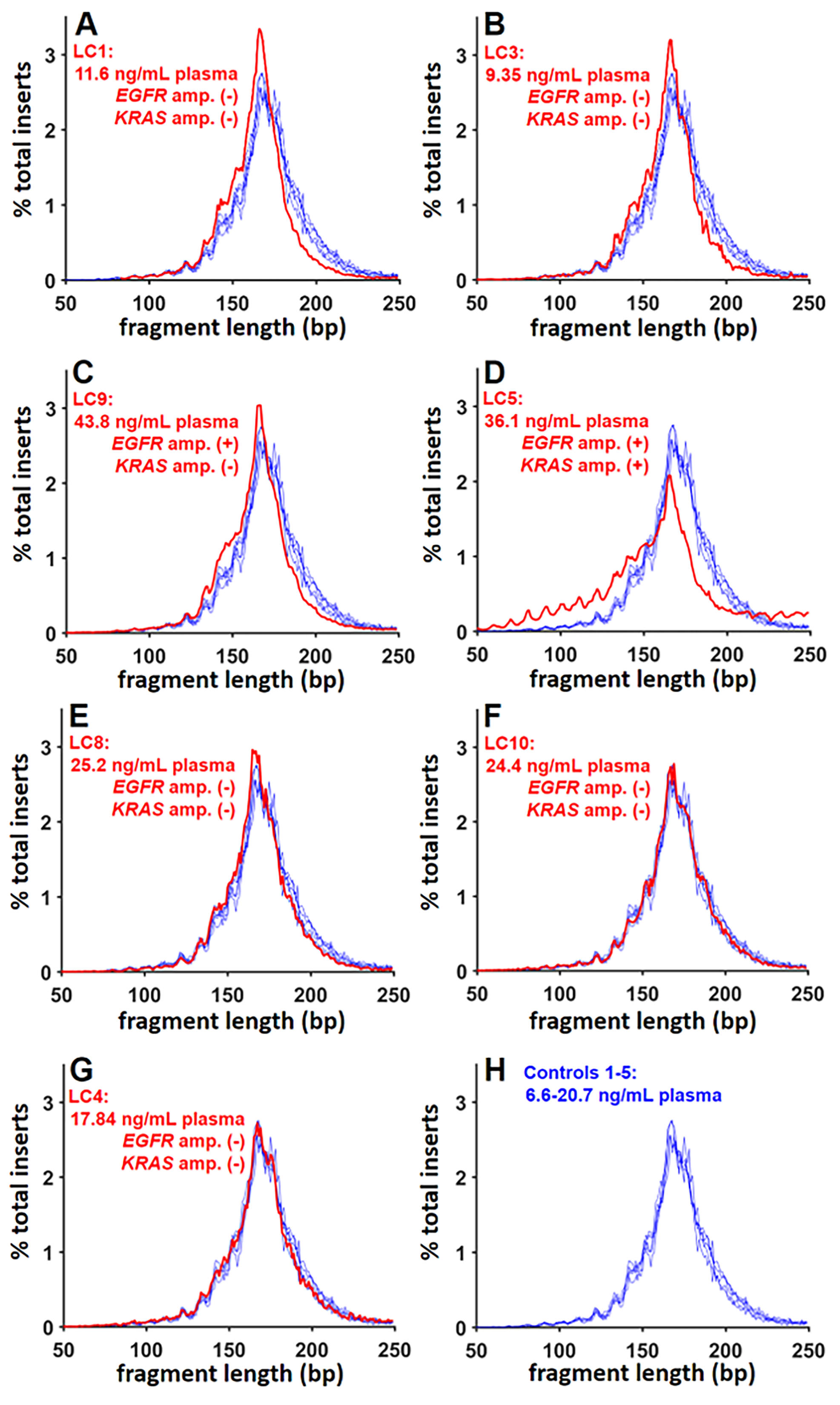

Supplement: S5 Fig — In A-G, the red line represents the fragment length distribution for a lung cancer patient, while the blue lines are the fragment length distribution for the five healthy controls. The plasma concentration of cell-free DNA and presence (+)/absence (-) of EGFR and KRAS amplifications are identified. In H, the fragment length distribution for only the healthy controls is shown along with the range of cell-free DNA plasma concentrations. (TIF) [file pgen.1006162.s005.tif]

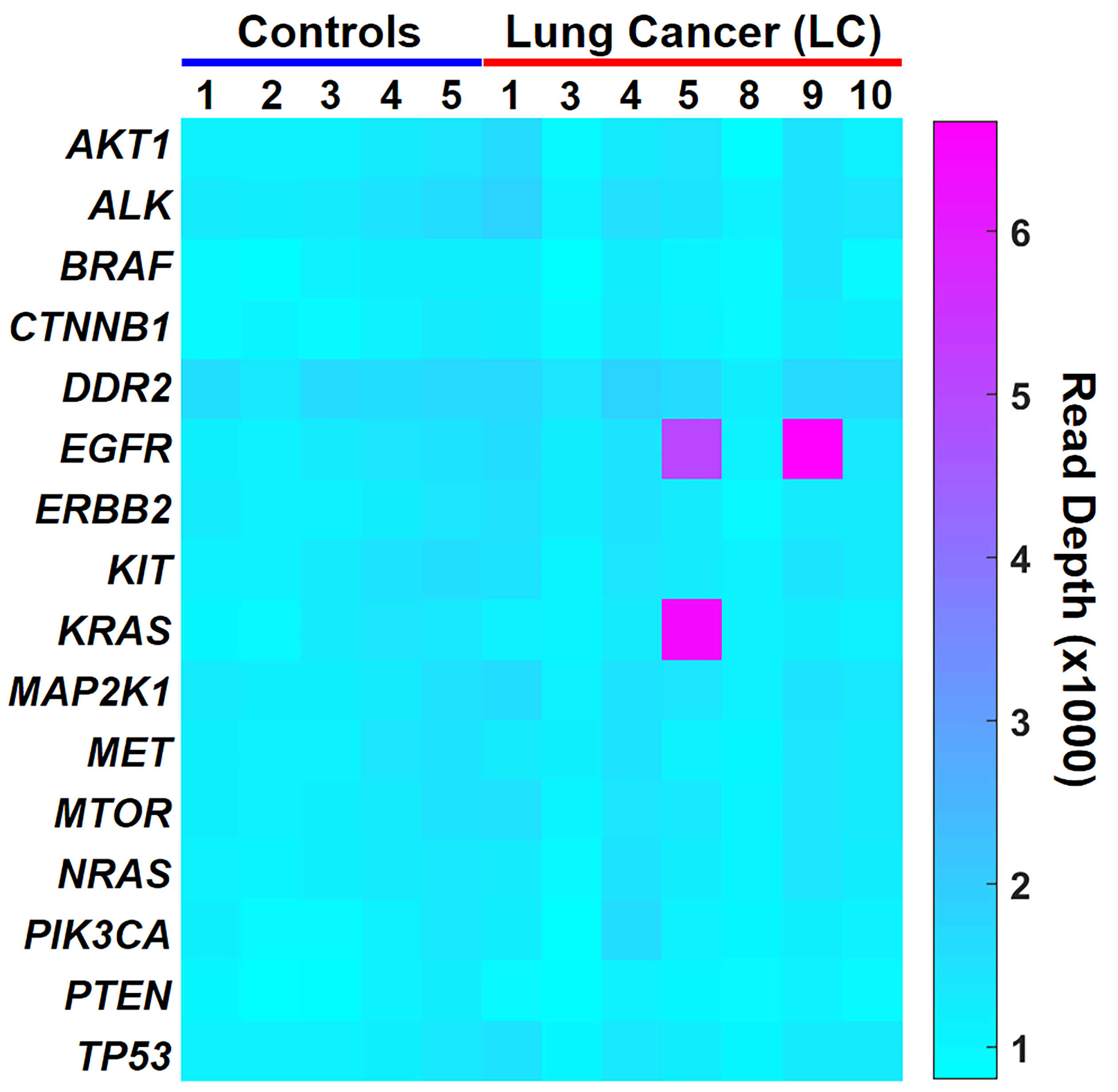

Supplement: S6 Fig — LC5 demonstrated amplification of EGFR and KRAS. LC9 had amplification of EGFR. No additional amplifications were evident. (TIF) [file pgen.1006162.s006.tif]

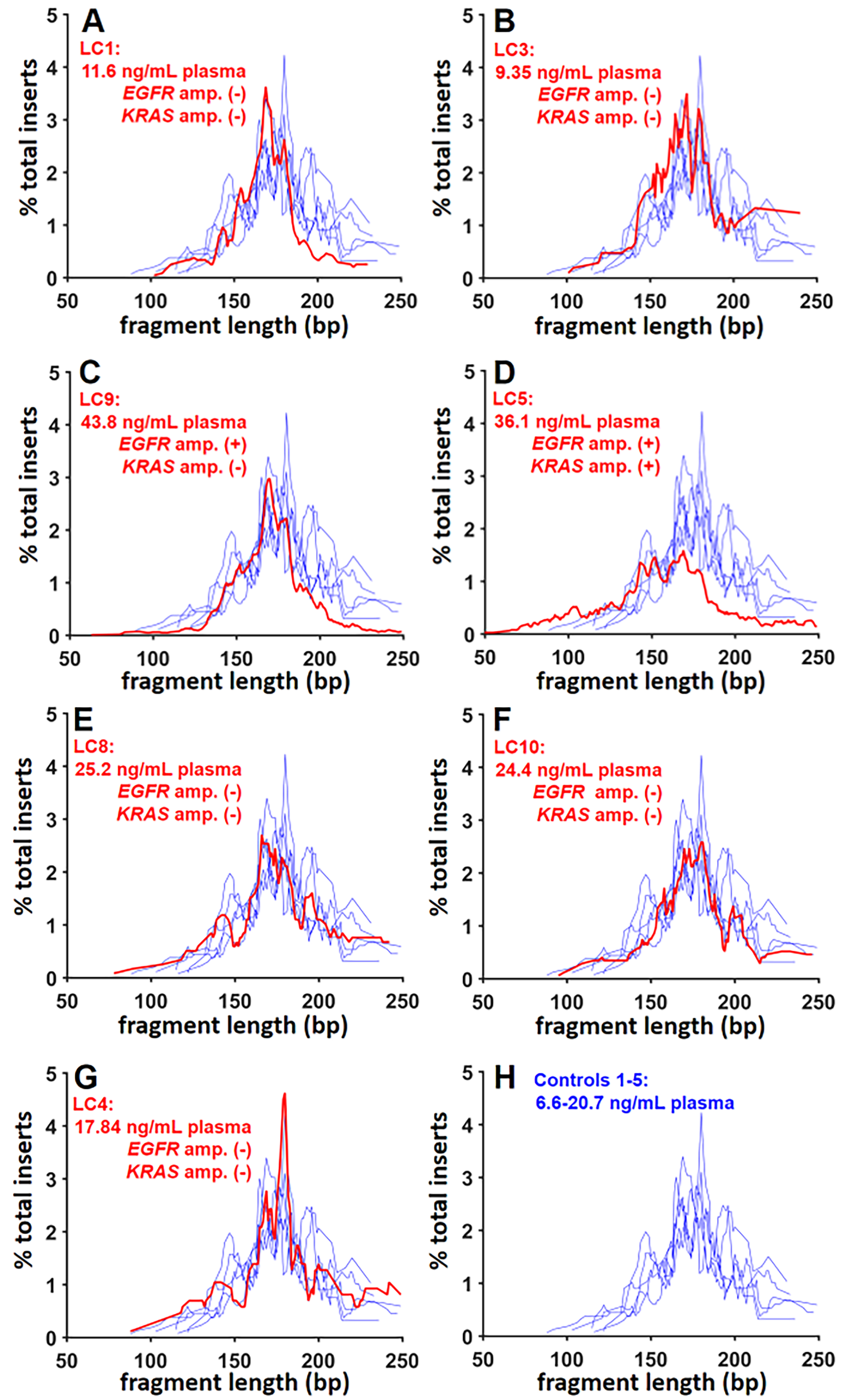

Supplement: S7 Fig — In A-G, the red line represents the fragment length distribution of the WT allele in a lung cancer patient, while the blue lines are the fragment length distribution of the WT allele in the five healthy controls. The plasma concentration of cell-free DNA and presence (+)/absence (-) of EGFR and KRAS amplifications are identified for the tumor patients. In H, the fragment length distribution of the WT allele for only the healthy controls is shown along with the range of cell-free DNA plasma concentrations. (TIF) [file pgen.1006162.s007.tif]

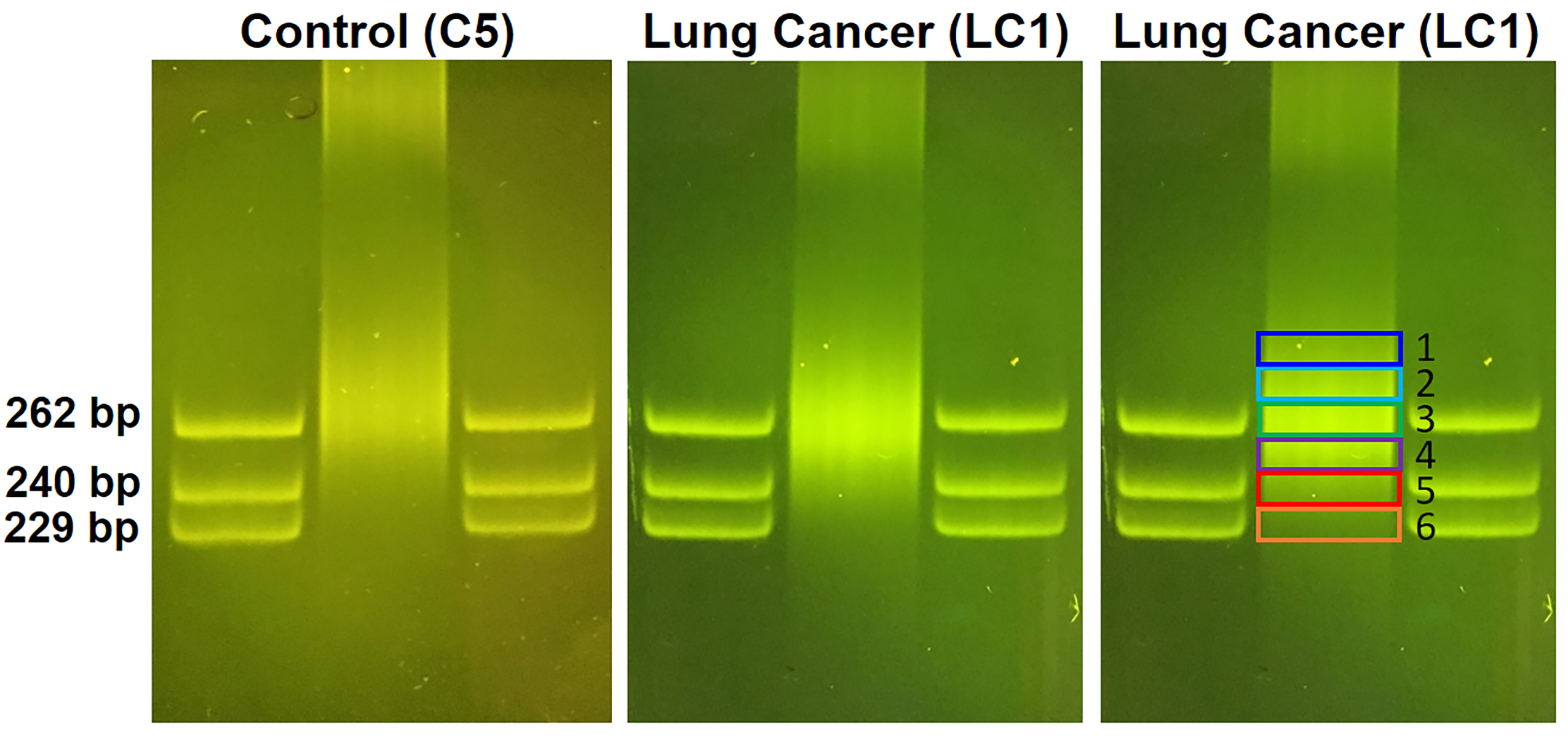

Supplement: S8 Fig — The ladder contained double-stranded DNA derived from phage lambda with lengths of 262 bp, 240 bp, and 229 bp. Using this ladder as a guide, six fractions were acquired from each library (far column). (TIF) [file pgen.1006162.s008.tif]

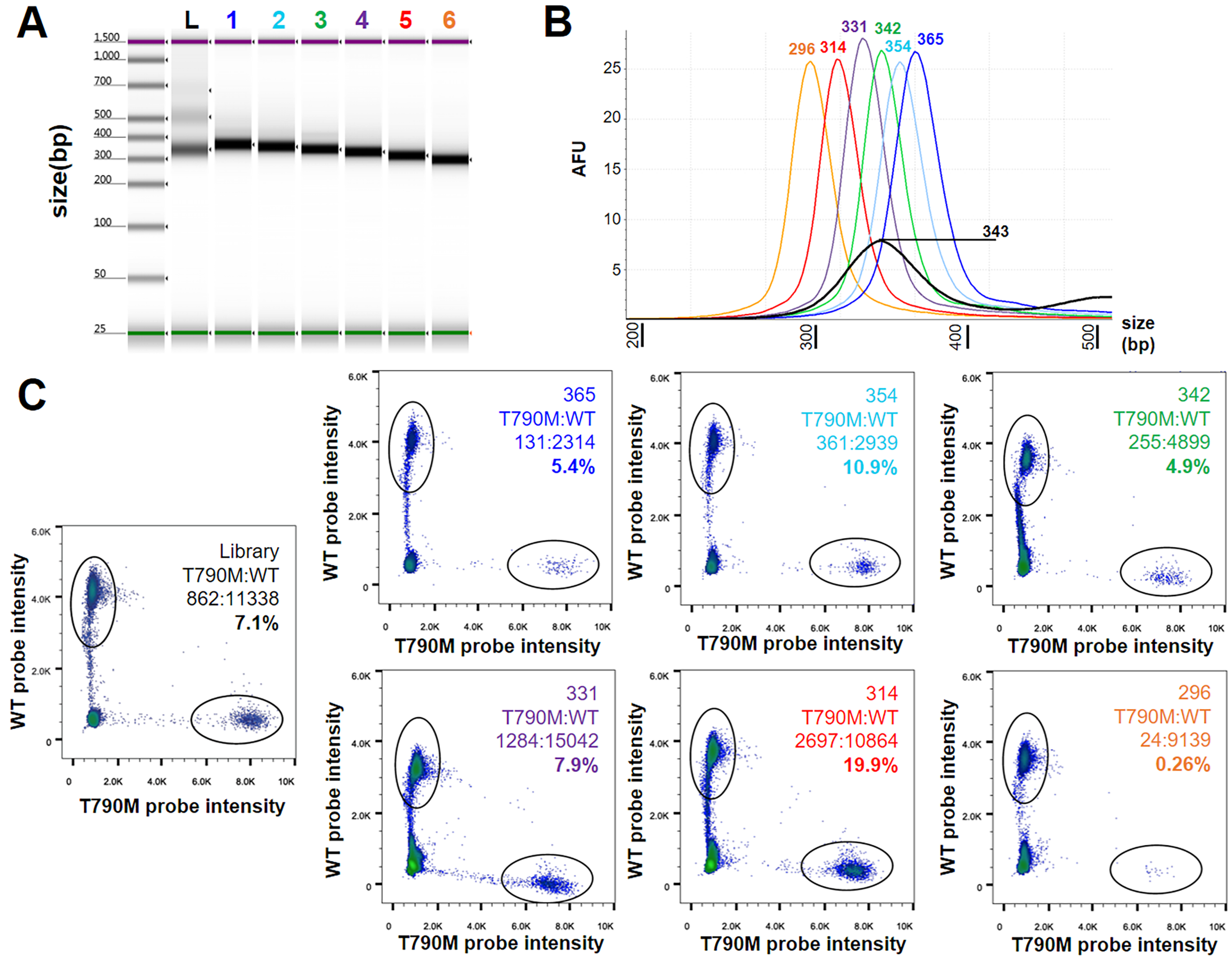

Supplement: S9 Fig — In A, the gel image of the library (L) and six fractions (colored numbers correspond to gel locations in S8 Fig) after amplification using the full-length adapter primers. In B, the fragment size distribution of each fraction (blue, light blue, green, purple, yellow, red line) and the library (black line) are shown. The fragment length associated with the peak is identified for each sample in a corresponding color. In C, the mutant allele frequency for the library and each fraction via digital droplet PCR are identified. In A-C, all colors indicate corresponding samples and are consistent with the colors used in S8 Fig. (TIF) [file pgen.1006162.s009.tif]

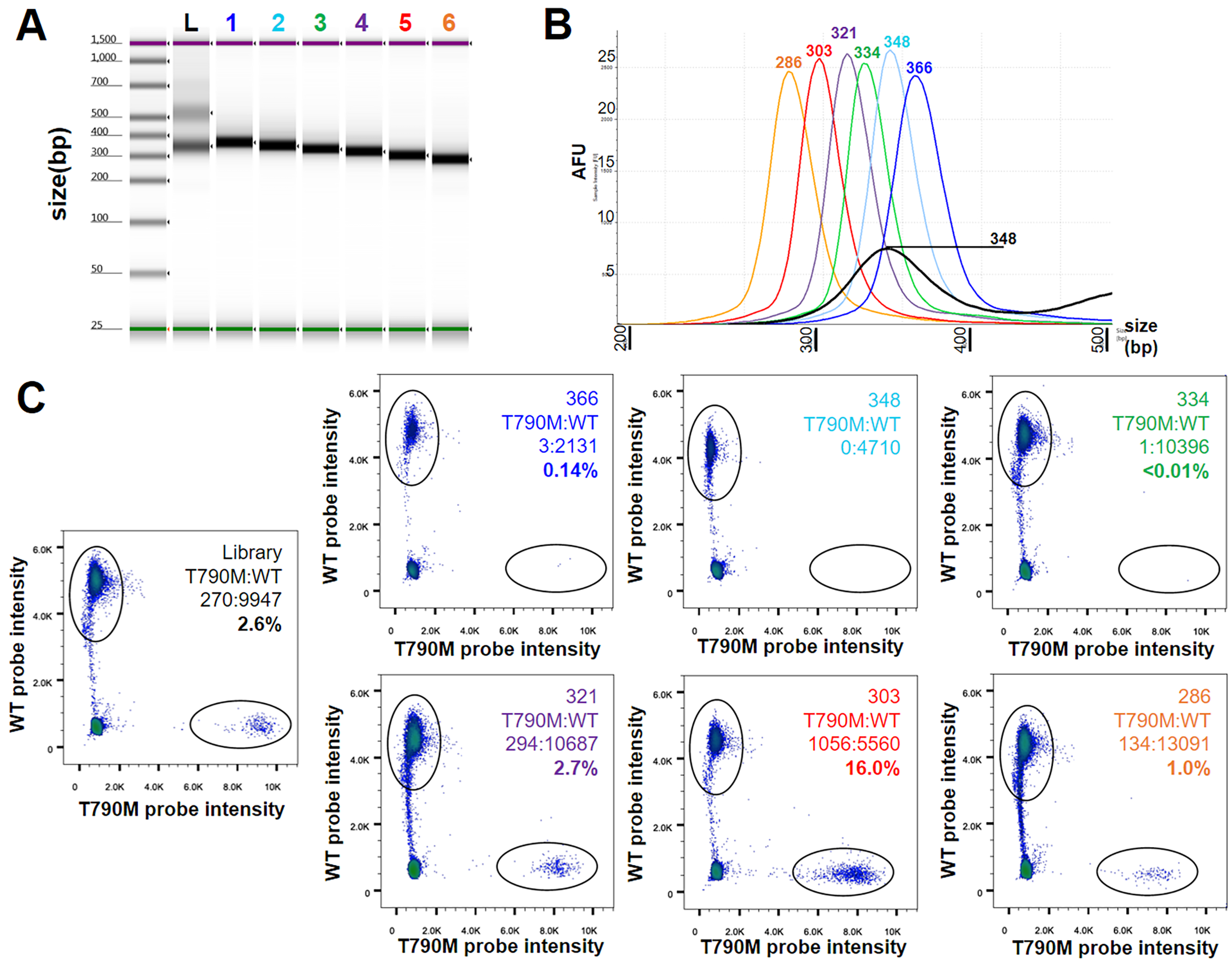

Supplement: S10 Fig — In A, the gel image of the library (L) and six fractions (colored numbers correspond to gel locations in S8 Fig) after amplification using the full-length adapter primers. In B, the fragment size distribution of each fraction (blue, light blue, green, purple, yellow, red line) and the library (black line) are shown. The fragment length associated with the peak is identified for each sample in a corresponding color. In C, the mutant allele frequency for the library and each fraction via digital droplet PCR are identified. In A-C, all colors indicate corresponding samples and are consistent with the colors used in S8 Fig. (TIF) [file pgen.1006162.s010.tif]

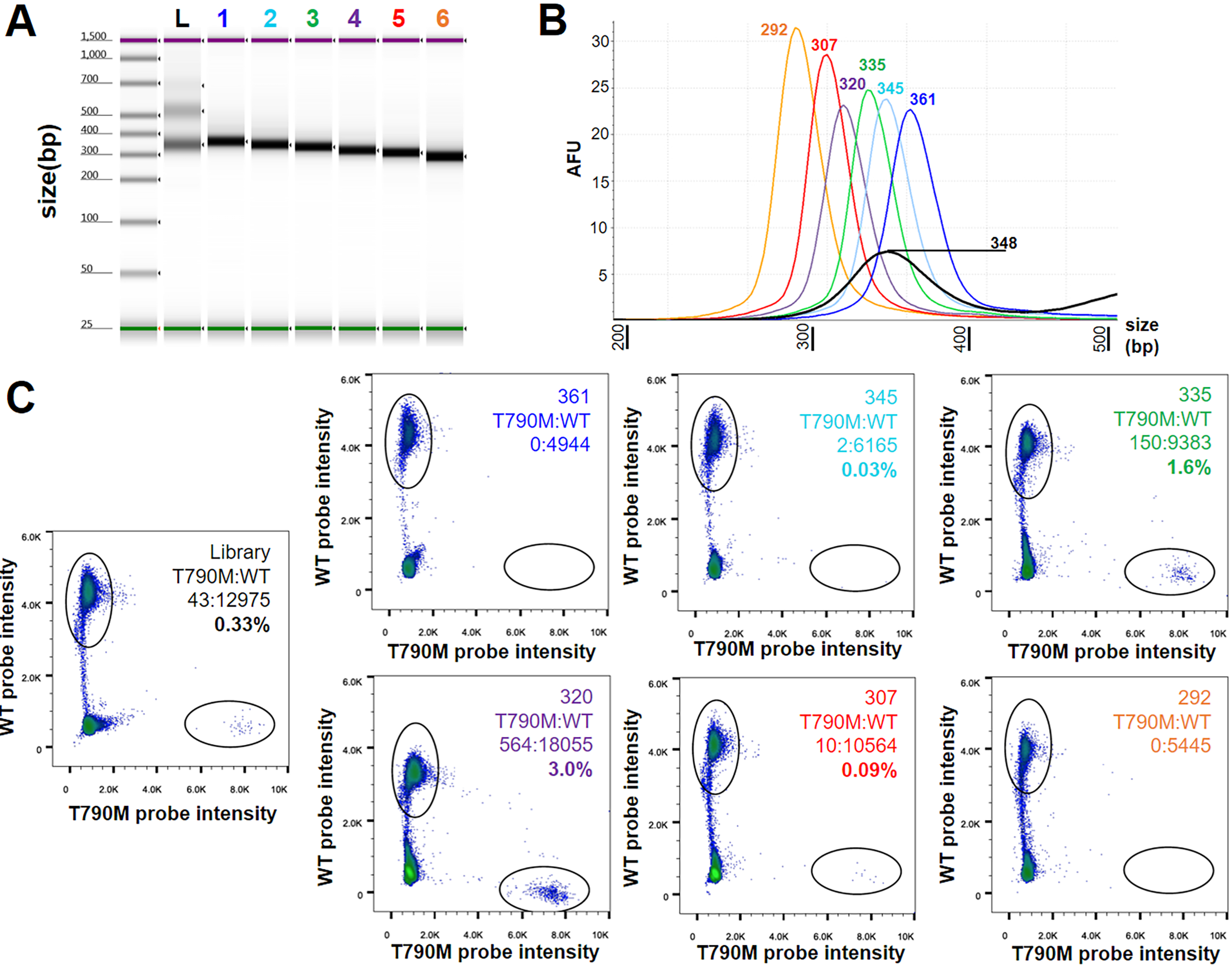

Supplement: S11 Fig — In A, the gel image of the library (L) and six fractions (colored numbers correspond to gel locations in S8 Fig) after amplification using the full-length adapter primers. In B, the fragment size distribution of each fraction (blue, light blue, green, purple, yellow, red line) and the library (black line) are shown. The fragment length associated with the peak is identified for each sample in a corresponding color. In C, the mutant allele frequency for the library and each fraction via digital droplet PCR are identified. In A-C, all colors indicate corresponding samples and are consistent with the colors used in S8 Fig. (TIF) [file pgen.1006162.s011.tif]

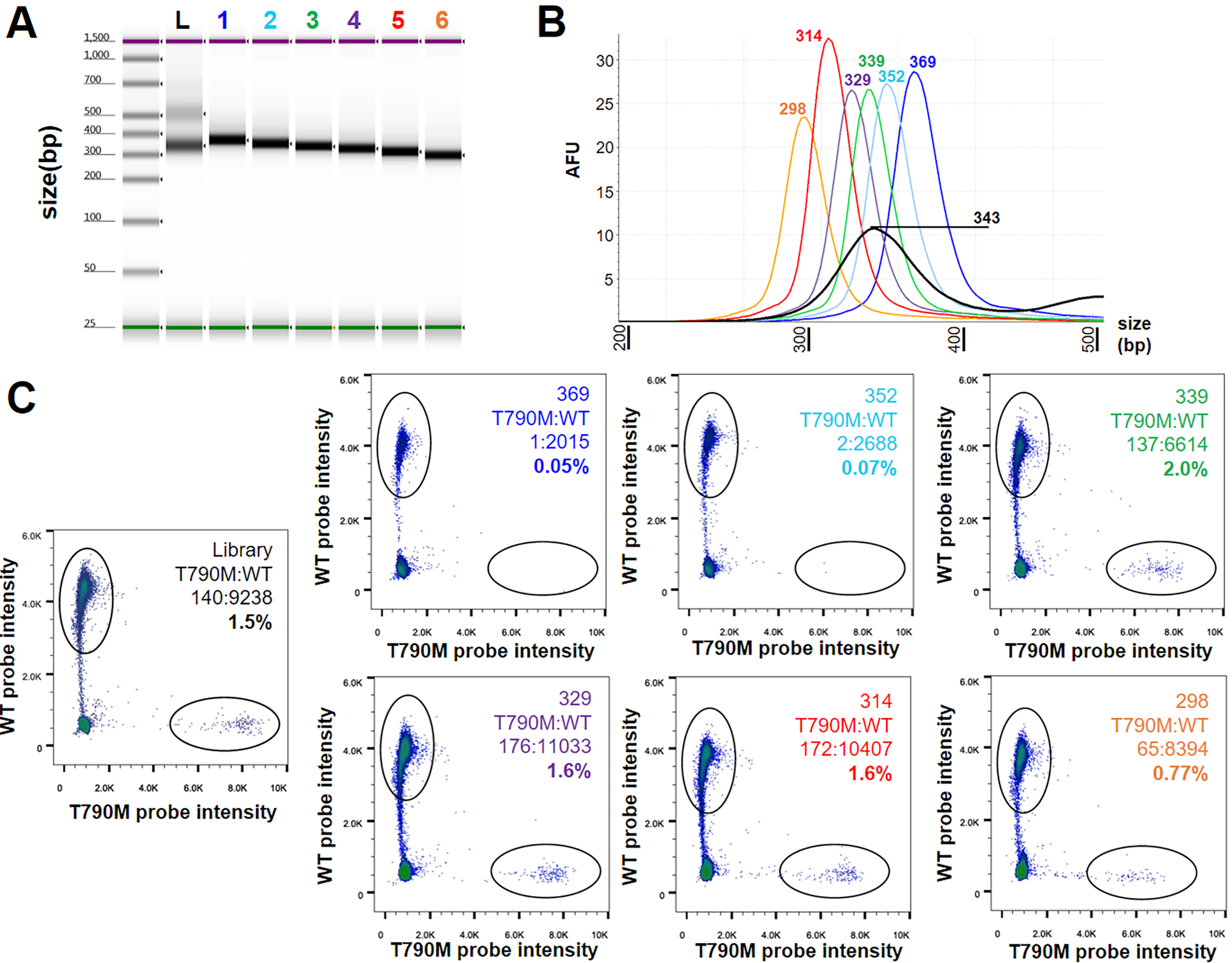

Supplement: S12 Fig — In A, the gel image of the library (L) and six fractions (colored numbers correspond to gel locations in S8 Fig) after amplification using the full-length adapter primers. In B, the fragment size distribution of each fraction (blue, light blue, green, purple, yellow, red line) and the library (black line) are shown. The fragment length associated with the peak is identified for each sample in a corresponding color. In C, the mutant allele frequency for the library and each fraction via digital droplet PCR are identified. In A-C, all colors indicate corresponding samples and are consistent with the colors used in S8 Fig. (TIF) [file pgen.1006162.s012.tif]

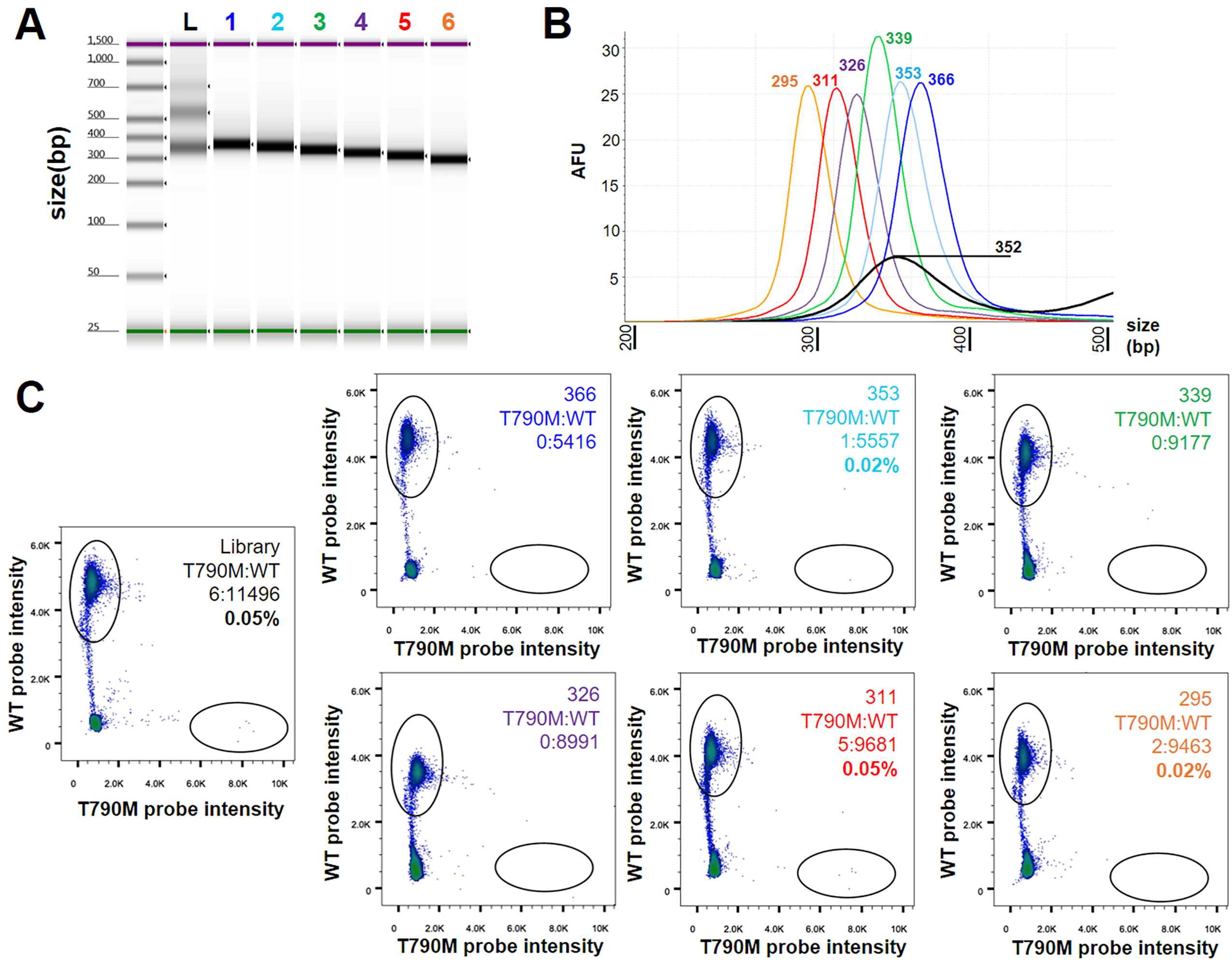

Supplement: S13 Fig — In A, the gel image of the library (L) and six fractions (colored numbers correspond to gel locations in S8 Fig) after amplification using the full-length adapter primers. In B, the fragment size distribution of each fraction (blue, light blue, green, purple, yellow, red line) and the library (black line) are shown. The fragment length associated with the peak is identified for each sample in a corresponding color. In C, the mutant allele frequency for the library and each fraction via digital droplet PCR are identified. In A-C, all colors indicate corresponding samples and are consistent with the colors used in S8 Fig. (TIF) [file pgen.1006162.s013.tif]

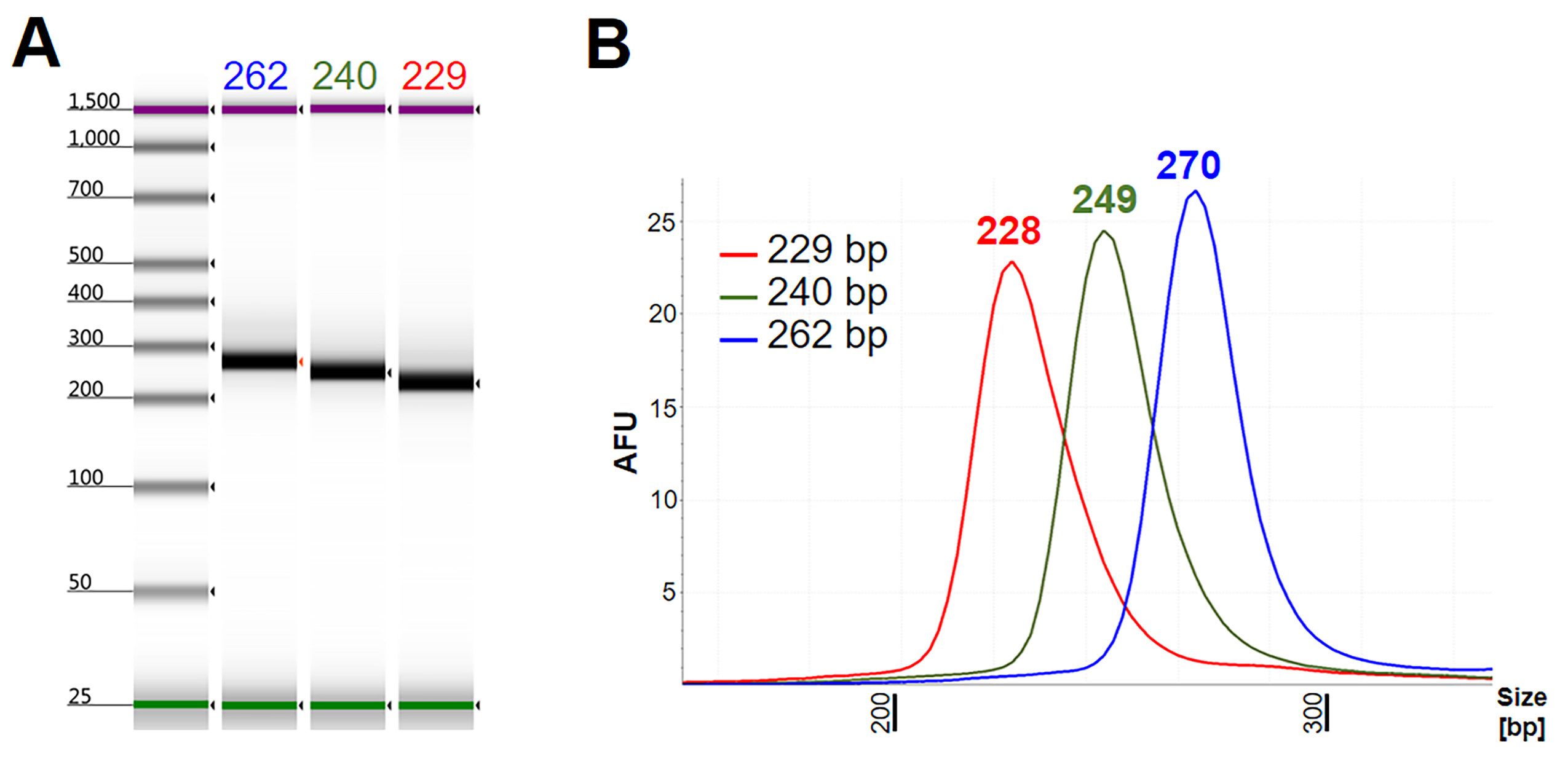

Supplement: S14 Fig — The ladder was constructed from phage lambda double-stranded DNA consisting of three lengths: 229 bp (red), 240 bp (blue), and 262 bp (magenta). An image of the gel is shown in A. In B, peak fragment length as measured by densitometry is identified for each element of the ladder above the corresponding peak. Estimation of fragment length by densitometry was susceptible to an overestimation up to ~10 bp. Relative differences in the ladder were better preserved during polyacrylamide gel electrophoresis (S8 Fig). (TIF) [file pgen.1006162.s014.tif]

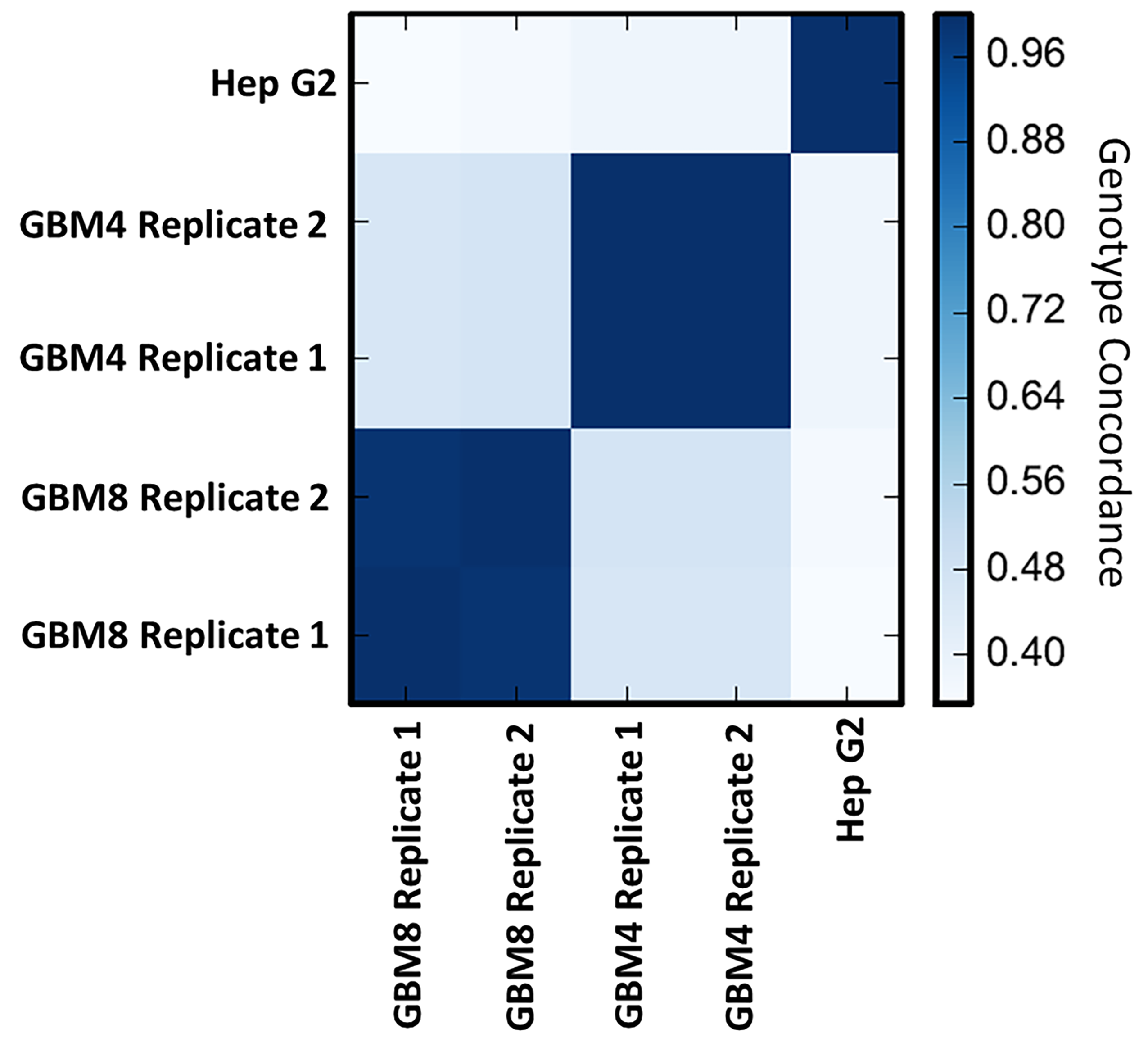

Supplement: S15 Fig — (TIF) [file pgen.1006162.s015.tif]
